# Aerosol-assisted route to low-E transparent conductive gallium-doped zinc oxide coatings from pre-organized and halogen-free precursor†

**DOI:** 10.1039/d0sc00502a

**Published:** 2020-04-27

**Authors:** Clara Sanchez-Perez, Sebastian C. Dixon, Jawwad A. Darr, Ivan P. Parkin, Claire J. Carmalt

**Affiliations:** University College London, Department of Chemistry 20 Gordon St London WC1H 0AJ UK c.j.carmalt@ucl.ac.uk

## Abstract

Thermal control in low-emission windows is achieved by the application of glazings, which are simultaneously optically transparent in the visible and reflective in the near-infrared (IR). This phenomenon is characteristic of coatings with wide optical band gaps that have high enough charge carrier concentrations for the material to interact with electromagnetic radiation in the IR region. While conventional low-E coatings are composed of sandwiched structures of oxides and thin Ag films or of fluorinated SnO_2_ coatings, ZnO-based glazing offers an environmentally stable and economical alternative with competitive optoelectronic properties. In this work, gallium-doped zinc oxide (GZO) coatings with properties for low-E coatings that exceed industrial standards (*T*_visible_ > 82%; *R*_2500 nm_ > 90%; *λ*_(plasma)_ = 1290 nm; *ρ* = 4.7 × 10^−4^ Ω cm; *R*_sh_ = 9.4 Ω·□^−1^) are deposited through a sustainable and environmentally friendly halogen-free deposition route from [Ga(acac)_3_] and a pre-organized zinc oxide precursor [EtZnO^i^Pr]_4_ (**1**) *via* single-pot aerosol-assisted chemical vapor deposition. GZO films are highly (002)-textured, smooth and compact without need of epitaxial growth. The method herein describes the synthesis of coatings with opto-electronic properties commonly achievable only through high-vacuum methods, and provides an alternative to the use of pyrophoric ZnEt_2_ and halogenated SnO_2_ coatings currently used in low-emission glazing and photovoltaic technology.

## Abstract 2

A pre-organised and halogen-free ZnO precursor is used to deposit GZO coatings *via* aerosol-assisted CVD with properties that exceed industrial standards for low-E coatings and photovoltaics.

## Graphical abstract

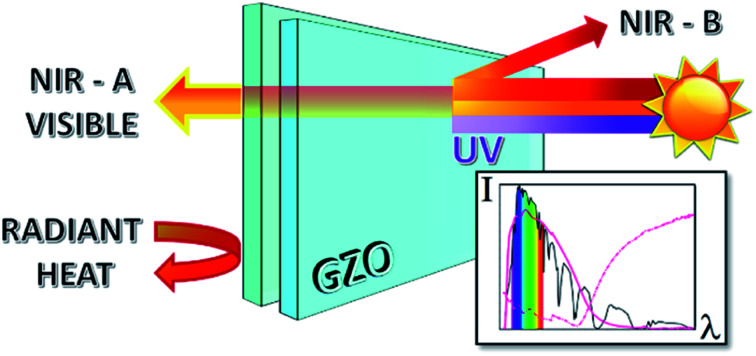

## Introduction

Thin films of gallium-doped zinc oxide (GZO) are a popular choice for transparent conducting oxide (TCO) applications. TCOs are indispensable in the modern world, spanning applications as coatings for low-emissivity windows and thin films in solar cells, flat panel displays and touch screens.^1^ The most commonly used materials for these applications are tin-doped indium oxide (ITO) or fluorine-doped tin oxide (FTO), however the scarcity of indium and the scrubbers required for deposition of halogen-doped TCOs increase considerably the final production cost of electronic devices.^2^ ZnO-based TCOs are considered amongst the most promising alternatives due to their comparable electronic and optical performance,^3–7^ while raw Zn is naturally abundant and cheap.^2,8^ Nominally undoped zinc oxide has inherent conductivity derived from the existence of native structural defects,^9–11^ controllable through promotion of non-stoichiometry and generation of shallow electron donors by varying the deposition environment^12,13^ and by performing post-deposition annealing under reducing conditions.^14–16^ However, in order to achieve the high reflectance in the mid and far IR necessary for low-emissivity applications^17,18^ and the low electrical resistivity needed for most optoelectronic purposes, it is necessary to introduce additional free electrons through extrinsic doping.^19,20^ The existence of surface plasmon resonance (SPR) effects has been reported for non-conventional plasmonic materials and TCOs such as doped ZnO and ITO show promising future^21,22^ as plasmonic materials in the near IR due to their metallic behaviour and smaller optical losses compared to Ag and Au.^22,23^ Although aluminium is a desirable dopant due to its low cost, it is highly reactive and leads to formation of a secondary oxide phase during film growth,^24^ segregation of dopant towards the film surface^25^ and poor humidity stability compared to gallium.^26^ Furthermore, the ionic radius of Ga^3+^ is closer to that of Zn^2+^ (Zn^2+^ = 0.74 Å; Ga^3+^ = 0.62 Å; Al^3+^ = 0.54 Å), leading to smaller dopant-induced crystal strain. Ga doping in ZnO films has also been reported to maximize the transparency of films over Al doping and therefore it is considered the preferable dopant for ZnO-based TCOs.^25^

The synthesis of GZO thin films requires consideration of several factors such as the choice of deposition method, zinc and gallium precursors, substrate, deposition temperature and dopant concentration, which are key for the optimization of optical and electrical properties of the thin films. Whilst several deposition methods are commonly used in industry, chemical vapor deposition (CVD) methods can produce highly dense and adhesive, pure thin films, with good control over film composition, coverage and uniformity on large scales.^27^ Furthermore, the possibility of overcoming volatility limitations using the solution-based method aerosol-assisted (AA)CVD opens a window for a wider range of precursors for ZnO-based TCOs.^28^ The optoelectronic properties of ZnO-based thin films are largely dependent on the film morphology and microstructure,^29^ and therefore an accurate optimization of deposition parameters within a particular methodology is necessary to lead to excellent device performances. In order to achieve excellent opto-electronic properties, a compact and near-to-monocrystalline microstructure is desirable. For that purpose, it is common to coerce crystal growth towards a singular crystallographic direction – self-texturing-, such as the polar plane (002) for ZnO wurtzite.^30^ The promotion of “self-textured” films grown preferentially towards this (002) direction is generally favoured in oxygen-rich conditions, which are the most commonly used in research and industry, and lead to tetrahedrally-coordinated ZnO. However, thin films grown in oxygen-deficient conditions exhibit preferential growth towards (110) and (101) planes, as commonly seen in dual-inlet systems using ZnEt_2_ and MeOH.^19^ Regardless of that, direct growth onto non-textured substrates such as glass usually allows growth towards several crystallographic directions, leading to polycrystalline samples with high surface roughness. A common way to promote exclusively (002) orientation is through epitaxial growth over a substrate like c-silicon and c-Al_2_O_3_, at the expense of reducing the cost-effectiveness of the deposition method.^31^ Hence, for any large are application such as low-e coatings, the use of epitaxial growth is not economical and therefore not employed. A wide variety of physical^24,32–35^ and chemical^15,25,29,36–44^ deposition methods have been used to develop ZnO based TCOs, all of which can produce thin films with visible transmittance over 80% in the visible region. Although PVD methods can generate ZnO-based films with electrical resistivity competitive with ITO (*ρ* ∼ 10^−4^ Ω cm),^30,45^ films grown using technologically more desirable techniques (such as ALD and CVD) tend to be polycrystalline in nature, which restricts mobility and carrier concentration values resulting in higher resistivity values (*ρ* ∼ 10^−2^ to 10^−3^ Ω cm).^15,25,29,31,36–44^ As solution-based methods require the use of organometallic precursors, their chemical nature can directly affect the formation path of the oxide. For example, the use of air-stable Zn precursors and Ga dopants with oxygen or nitrogen – saturated 1^st^ coordination sphere ligands (acetylacetonates, acetates, nitrates, *etc.*) *via* single-inlet methods, namely AACVD,^25,29,42–44^ sol–gel^38–40^ and spray pyrolysis^41,46^ have been shown to generate largely (002)-oriented films with resistivity values in the order of 10^−1^ to 10^−3^ Ω cm. In contrast, reaction of corrosive precursors and dopants (diethyl zinc, triethylgallium, *etc.*) with alcohols/water using dual-source CVD methods produce films with mainly (100) and (101) preferential orientation while achieving minimum resistivity values of 10^−4^ Ω cm for both ZnO^19^ and GZO.^35^ The substantial differences found using the aforementioned two types of precursors/dopants raise the question of whether a relationship exists between the metal 1^st^ coordination sphere of a precursor – which determines the oxygen-rich/poor conditions of growth around the Zn atoms rather than the carrier media-, the promoted crystallization planes and the electrical properties of the resulting GZO thin films. As conduction in ZnO is anisotropic, a relation between crystallization planes and conductivity seems likely.

From a manufacturing perspective, the use of non-hazardous and environmentally friendly precursors in CVD processes is of great importance for large scale applications as risks associated with highly reacting precursors should be minimised.^27^ Numerous alternative precursors have been investigated, such as [Zn(OAc)_2_],^47^ [Zn(acac)_2_],^25,43^ Zn(thd)_2_,^48^ Zn(hfac)_2_(amine),^49^ Zn(tmp)_2_,^50^ Zn(TTA)_2_TMEDA,^51^*etc.* to avoid the handling problems associated with alkylzinc reagents. Such advances in the field of precursor design for ZnO thin films tackle stability issues and associated hazards, however progress has been driven mainly by volatility requirements, which restricts the scope of precursor selection. In addition, most chelating ligands used in these precursors contain large quantities of carbon and nitrogen (up to 80–90% of precursor mass), often leading to significant carbon contamination in detriment to the optoelectronic properties of the resulting films.^31,52,53^ Hence, an overall effective precursor for CVD should have ligands with low hydrocarbon content relative to the total mass of the precursor, should undergo a clean decomposition process at low enough temperature to ensure the deposition of crystalline films and should have low oxygen saturation around Zn atoms.^28^

The successful use of tetrameric heterocubane-like alkylzinc alkoxides [MeZnOR]_4_ (R = ^i^Pr, ^*t*^Bu) as single-source MOCVD precursors for ZnO thin films with low carbon contamination was reported to undergo a β-hydrogen elimination process through the formation of a cyclic 6-member transition state.^54^ These films, however, were described to be dark, highly resistive, and to exhibit very rough surface morphology. Alkylzinc alkoxides of the same type [R_1_ZnOR_2_]_4_ have been reported as excellent precursors for crystalline nanostructured oxygen-deficient ZnO nanoparticles *via* solution methods,^55–57^ as a result of the cubic {Zn–O}_4_ central core promoting growth in an elongating direction after dimerization.^55^ These alkoxides have a pre-organized ZnO structure that could generate highly oriented ZnO films, with desirable morphological features required for high-end TCO coatings. However, in their study Auld *et al.* mention that the oxygen in these precursors is not sufficient for the complete oxidation of zinc and the optoelectronic properties of the deposited films are rather poor, hence an additional oxygen source would be necessary during deposition.^54^ The use of AACVD method can drastically increase the potential of these moisture/air sensitive alkylzinc alkoxides as precursors for high-end TCOs since the anhydrous alcohol ROH can simultaneously act as carrying solvent with low carbon content and oxygen source during depositions.

The alkylzinc alkoxide molecular precursor [EtZnO^i^Pr]_4_ (**1**) has been evaluated as an AACVD precursor for highly directional ZnO-based materials. It possesses the desired Zn/O ratio of 1 : 1 in its cubane-like core structure, it can undergo a clean decomposition path through β-hydrogen elimination of volatile by-products, and it is expected to promote oxygen-deficiencies due to the possibility of acetone elimination associated with the ^i^Pr–O moieties through a β-hydride transfer process.^58^ All of these factors make this precursor an excellent candidate for the synthesis of high-quality ZnO-based coatings for opto-electronic applications without the need of epitaxial substrates. Furthermore, its stability in the presence of 2-propanol and [Ga(acac)_3_] allows for a simple single-inlet injection setup to generate GZO coatings with high growth rate (70–100 nm min^−1^). Finally, the use of halogen-free synthetic routes allows for environmentally friendly and industrially sustainable deposition of TCO coatings without the need of scrubbers.

## Results and discussion

### Synthesis and physical characterisation

**Scheme 1:**
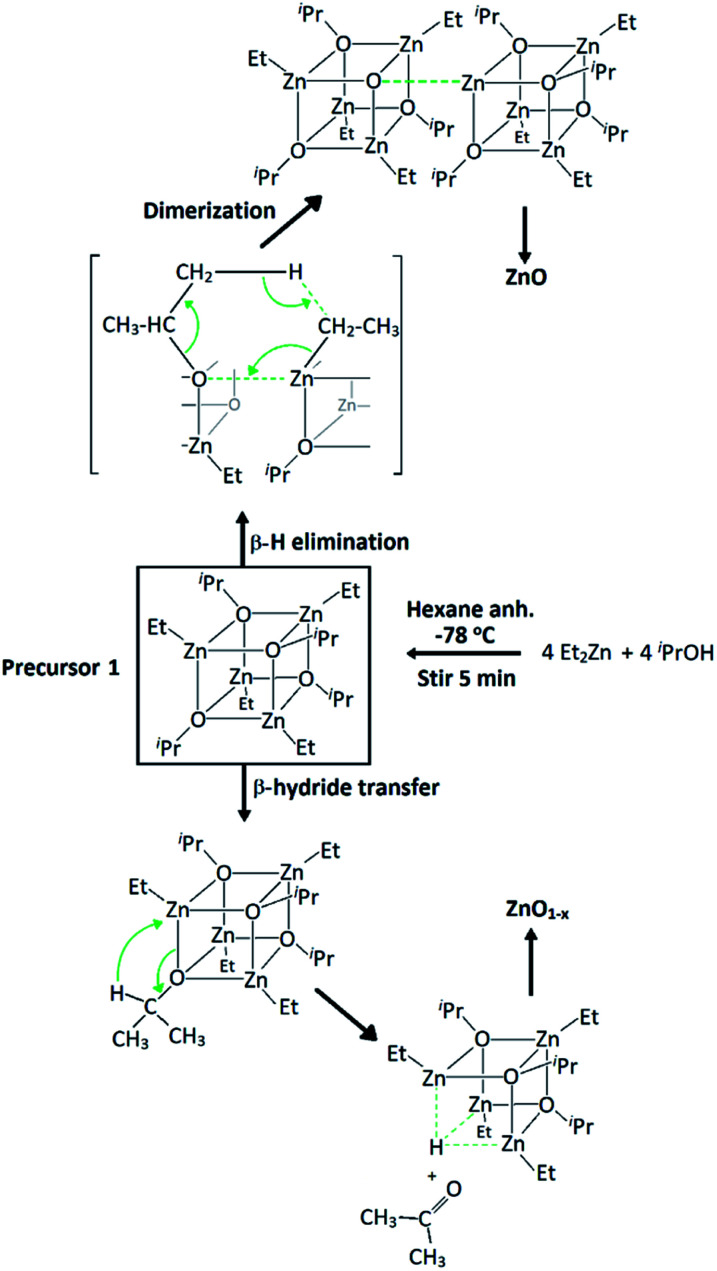
Synthesis of precursor [EtZnO^i^Pr]_4_ (**1**) and decomposition paths for the formation of zinc oxide through β-hydrogen elimination and β-hydride transfer.

**Fig. 1:**
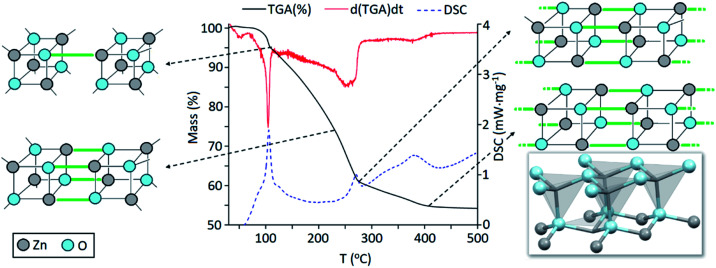
Thermogravimetric analysis (TGA, black line), differential calorimetry (DSC, dashed blue line) and 1^st^ derivative of mass loss over time (red line) or the thermal decomposition of precursor [EtZnO^i^Pr]_4_ (**1**) under helium. The crystal structure of ZnO (wurtzite) is reproduced from ICSD.^60^

Precursor **1** [EtZnO^i^Pr]_4_ was synthesised by addition of ZnEt_2_ and 2-propanol in anhydrous hexane at −78 °C, and isolated *in vacuo* for characterisation (experimental section). Thermogravimetric analysis (TGA) and differential scanning calorimetry (DSC) measurements of **1** were carried out both under ambient and inert conditions. The possibility of two different elimination processes *via* β-H elimination^55^ and β-H migration^58^ (Scheme 1) enables the formation of oxygen-deficient thin films. TGA of precursor **1** under helium (Fig. 1) exhibits a first sharp endothermic step at 105 °C (6% total mass loss), a second and third step at 230 °C (23.5%) and at 275 °C (39%), followed by a final decomposition step at 415 °C accounting for a total loss of 45.5% (theoretical 46%), which is in good agreement with literature values.^56,59^ The data appears to be consistent with a decomposition mechanism involving a dimerization process (step 1), supported by the formation of an insoluble white precipitate. Further reaction of the organic ligands through β-hydrogen elimination for crystal growth in a longitudinal direction towards ZnO wurtzite – tetrahedra of {ZnO_4_} sharing corners – is supported by TGA data, as previously suggested by Boyle *et al.*^55^ Thermogravimetric studies of related precursors propose a similar decomposition path through a dimeric intermediate.^56^

**Fig. 2:**
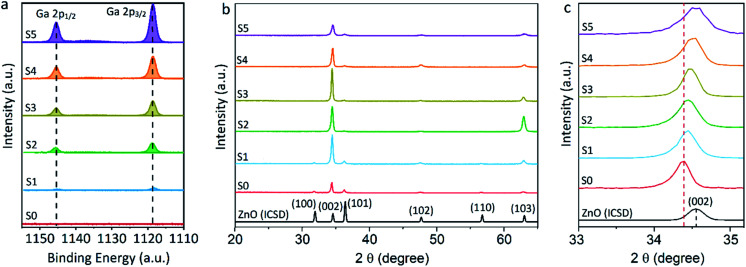
X-ray photoelectron spectroscopy (XPS) scans of (a) Ga 2p peaks for ZnO and GZO thin films with 0.7–7.0 at% of Ga and (b) X-ray diffraction (GIXRD) patterns in the range 2*θ* = 20–66° of undoped ZnO (S0) and gallium-doped ZnO (S1–S5) thin films (coloured lines) and PXRD standard of wurtzite ZnO (black line) and (c) selected area of (002) planes of X-ray diffraction (GIXRD) normalised patterns.

**Fig. 3:**
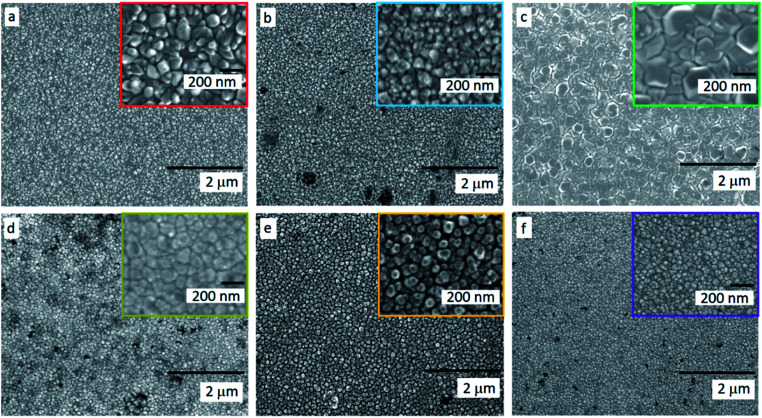
Scanning electron microscopy (SEM) micrographs of (a) undoped ZnO (S0) and gallium doped ZnO films (b) S1, (c) S2, (d) S3, (e) S4 and (f) S5. High resolution images of samples are embedded in each image.

**Fig. 4:**
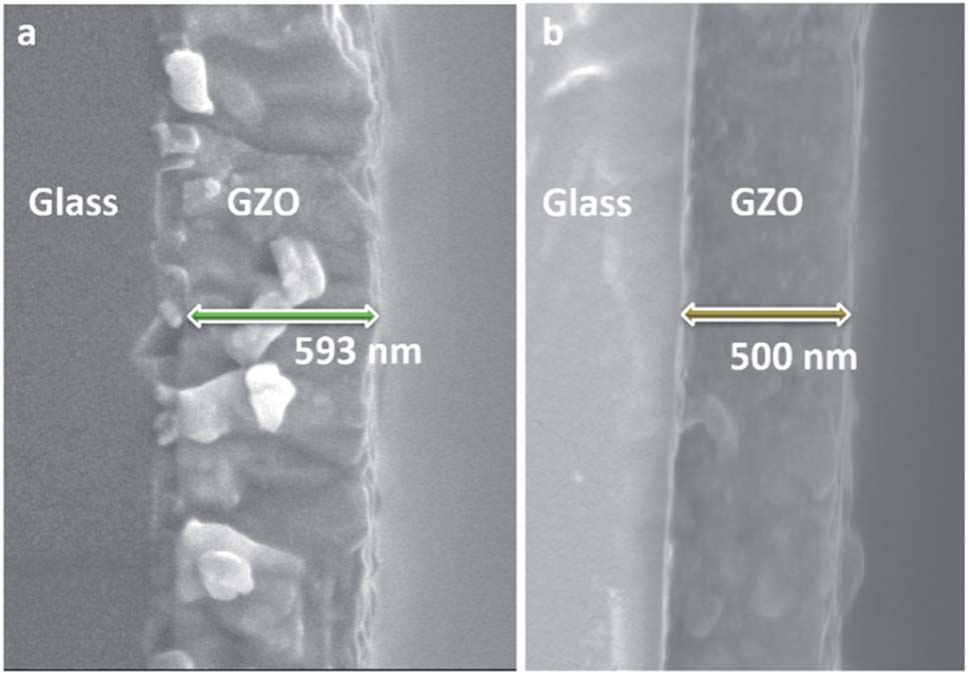
Cross-sectional SEM images of GZO thin films (a) S2 and (b) S3.

Equivalent studies of precursor **1** in air exhibited a fast decomposition at 95 °C, which is not unexpected in the presence of traces of moisture (Fig. S1†).^54^ Increasing amounts of [Ga(acac)_3_] (1–16% mol) were added to a precursor solution of **1** in anhydrous 2-propanol (15 mL). AACVD of the solutions were carried out at 425 °C under an inert atmospheric pressure of N_2_. Uniform transparent films were deposited in all cases, fully covering the substrate. X-ray photoelectron spectroscopy (XPS) has been used to detect the chemical nature of all films. XPS was performed over 3 levels of etching (200 s each) to evaluate the presence of elements in each film, and their chemical nature. The survey spectra indicated the presence of carbon, oxygen, zinc and gallium in all films. Carbon signals were significantly reduced upon argon sputtering (<2%), indicating that contamination was mainly limited to the surface. Zn peak positions (2p_3/2_ = 1021.8 ± 0.2 eV, *Δ* = 27 eV) and O peak positions (O 1s = 530.8 ± 0.2 eV) confirm the presence of Zn^2+^ and O^2−^ in a ZnO environment.^62,63^ Ga 2p peak positions (2p_3/2_ = 1118.7 ± 0.2 eV, *Δ* = 27 eV, Fig. 2a) coincide with those of Ga^3+^ in a Ga_2_O_3_ environment while no Ga^0^ is obviously present (2p_3/2_ = 1116.3 eV).^64^ Deconvolution of the Ga 3d environments supports the existence of Ga^3+^ (Ga 3d_5/2_ = 19.85 ± 0.2 eV, *Δ* = 0.46 eV) in the Ga_2_O_3_ environment.^65^ The existence of a secondary phase of gallium oxyhydroxide – likely due to surface hydration – cannot be ruled out, which would lie within the same energy range (Ga 3d_5/2_ = 19.90 ± 0.2 eV).^66^ No Ga^0^ (Ga 3d_5/2_ = 18.00 ± 0.2 eV – sharp signal expected) is found in the surface or after etching^64,67^ (Fig. S2 and S3†). Elemental analysis of films was carried out using X-ray fluorescence (XRF) spectroscopy. The efficiency of dopant incorporation was reported to decrease with increasing amount of gallium, and it was calculated to be in the range 44–78% based on the quantity of gallium incorporated in the films, which is proportional but not equivalent to the ratio of Zn and Ga precursors (Table 1). Crystal structures of ZnO and GZO films were analysed by Grazing Incident X-ray Diffraction (GIXRD). Reflection signals for all XRD patterns show alignment to peaks typical for hexagonal wurtzite ZnO (*P*6_3_*mc*-186)^68^ (Fig. 2b). Unit cell parameters are in strong agreement with accepted literature experimental values (*a* = 3.251 Å and *c* = 5.202 Å) (Table 1).^60^ Texture coefficients (TC(*hkl*)) for all peaks were calculated from their intensities relative to each other and to the standard powder pattern (ICSD 29272), as per eqn (S1) in the ESI.† The calculated TC(*hkl*) are plotted in Fig. S4† against the film at% Ga concentrations. The variation in TC(*hkl*) with increased doping follows a clear preference for *c*-axis orientation as indicated by the consistently strong texture coefficient for the (002) plane, which has often been reported for ZnO films grown *via* CVD methods.^52,69^ The introduction of a dopant in the ZnO structure has been recorded to alter the surface energy of specific crystallographic planes,^70–72^ promoting highly oriented morphological features mainly towards the (002) plane.^73^ While at lower at% Ga doping amounts (002)-oriented growth still dominates the patterns, crystal growth occurs at some degree towards all other planes, resulting in the emergence of other peaks in the pattern (Fig. 2b). At doping levels in the range ∼2–4 at% Ga maximum preferential orientation towards the (002) plane was achieved, with only secondary growth towards the (103) plane. At doping levels higher than 3.8 at% Ga growth was promoted towards (101), (102) and (103) planes, but restricted for (100) and (110). The lattice parameters of ZnO and GZO thin films indicate a slight unit cell decrease along the *c*-axis with increasing doping level (Table 1), which can be explained by the increasing substitution of Zn^2+^ with a dopant with a smaller ionic radius.^74^ The substitution of Zn^2+^ by the relatively smaller Ga^3+^ ions is expected to induce changes in crystallinity due to the introduction of structural disorders and defects,^75^ which ultimately leads to slight shift of the (002) peak position, previously calculated to be Δ2*θ* = 0.15°,^76^ towards higher Bragg's angle relative to that of undoped ZnO. The (002) diffraction peak for ZnO thin film (2*θ* = 34.378°) is slightly smaller than that of ZnO in bulk (2*θ* = 34.550°), which has been previously reported for thin films and attributed to a strong in-plane alignment with a close packing of crystal grains.^77^ The shift detected for the (002) plane increases with increasing amount of at% Ga (Fig. 2c), and its deviation from that of pure ZnO thin film is in good agreement with the calculated values by Kim *et. al.*^76^ (See Table 1). The addition of Ga to the ZnO structure produce an increase in crystallinity until the doping “solubility limit” (3.8 at% Ga), followed by a decrease in crystallinity and mean grain size, as GIXRD patterns of S4 and S5 exhibit clear wider FWHM in Fig. 2c. Scanning electron microscopy (SEM) showed that pure ZnO was composed of relatively uniform 100–150 nm wide wedge-like grains. This morphology is consistent with a weak promotion of growth in near *a*-axis orientations during crystallization (Fig. 3b).^42^ At low doping level (sample S1) the film surface morphology remained similar but experienced a decrease in crystallite size to 50–100 nm (Fig. 3b). For higher levels of doping (sample S2, S3) the film surface exhibited shallow wedge-like flat crystallites with hexagonal faces (Fig. 3c and d). This morphology appeared to be due to growth promotion mainly towards the (002) plane and to a lesser degree towards the (103) plane, and therefore films are compact and smooth (Fig. 4). Thin film S3 (Fig. 4b) exhibited particularly compact featureless cross sectional morphology with a very smooth surface, usually only detected in GZO films grown using PVD methods.^24,33,34,78^ Nevertheless, when the doping saturation point of ZnO is reached, doping efficiency dramatically decreases and dopant impurities tend to segregate towards grain boundaries and boost grain boundary movement, which results in both grain size and crystallinity reduction,^79^ which is visible apparent in the obtained films. This is a commonly observed phenomenon in n-type TCOs with high charge carrier density and is related to an increasing low-energy surface plasmon resonance (SPR) effect.^80^

**Table 1:** Doping amounts of Ga (at% Ga), Ga incorporation efficiency (Ga_eff_), film thickness (*d*), (002) texture coefficient (TC(002)), (002) diffraction peak (2*θ*(002)) and lattice parameters (GSAS/EXPGUI)^61^ for ZnO and GZO films with increasing at% of Ga

Sample ID	at% Ga [%]	Ga_eff_ [%]	*d* [nm]	TC (002)	2*θ* (002) [°]	*a* [Å]	*c* [Å]
S0	0	—	592	3.72	34.390	3.2536(3)	5.2061(4)
S1	0.7	78	520	4.45	34.446	3.2515(6)	5.2046(3)
S2	1.8	52	593	3.26	34.453	3.2460(4)	5.2028(2)
S3	3.8	50	500	4.84	34.472	3.2555(5)	5.2020(2)
S4	5.8	48	530	4.30	34.516	3.2502(6)	5.2019(3)
S5	7.0	44	560	3.95	34.547	3.2505(5)	5.1858(5)

**Fig. 5:**
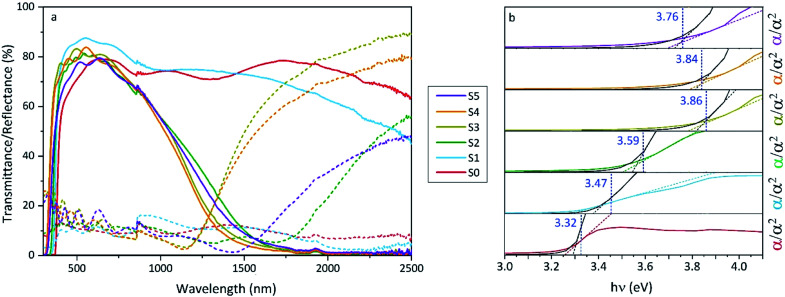
(a) Optical transmission and reflection spectra and (b) corrected Tauc^76^ plots of undoped ZnO (S0) and gallium-doped ZnO thin films with increasing at% of Ga (S1–S5). Transmittance/reflectance crossover in the spectra shows surface plasmon resonances for S2 (1566 nm), S3 (1292 nm), S4 (1284 nm) and S5 (1578).

**Fig. 6:**
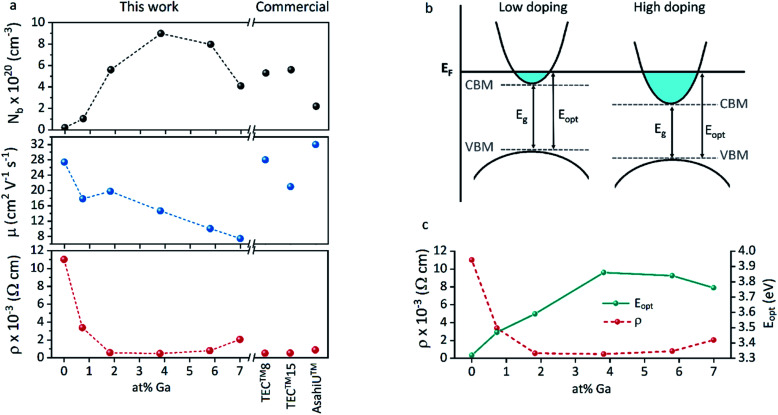
(a) Carrier concentration (*N*_b_), Hall mobility (*μ*) and resistivity (*ρ*) for ZnO and GZO thin films with different at% Ga. (b) Schematic representation of the electrical and optical (Moss–Burstein) band gap shifts and (c) comparative trends in resistivity (*ρ*) and film optical band gap (*E*_opt_) with increasing doping concentration.

The increase of charge carrier density with respect to ZnO is directly correlated with increased infrared reflectivity, however a further increase in doping beyond the optimum level reduction in the charge carrier density, reducing the infrared reflectivity. Optically transparent materials in the visible region of the spectrum with high reflectivity in the near-IR are of great relevance for solar energy applications and low-emissivity coatings, and are relatively uncommon for ZnO-based materials from CVD methods.^81^ GZO can be effectively doped to achieve a high carrier concentration despite the relatively small solid solubility limit of Ga in ZnO. The optical properties of GZO are therefore dependence on the doping concentration, especially when doped as close to the solid-solubility limit as possible.^82^ The GZO films show that by increasing the at% Ga doping in the range below the optimum value, the crossover frequency and the optical loss become lower, which can be respectively rationalised because of the increase of carrier concentration and the improvement in film crystallinity.^76^ The plasmon resonance wavelengths of the GZO thin films S2–S5 suggest high charge carrier values in the order of ∼10^21^ cm^−3^ (plotted separately in Fig. S5†) with values that exceed standards for commercial FTO coatings, and are particularly low for S3 and S4 (∼1250 nm) (Fig. 5a).^18,83^ Calculation of the optical band gaps for pure ZnO and GZO thin films was performed using a corrected Tauc plot^84^ for polycrystalline TCOs by Dolgonos *et al.*^85^ (Fig. 5b) and the corresponding band gap values are listed in Table 2. The band gap of pure ZnO film (3.32 eV) was gradually enhanced with Ga dopant through the addition of free electrons from donor Ga^3+^ ions to the bottom level of the conduction band, which leads to an increase in the Fermi level (Burstein–Moss effect)^86^ to a maximum value of *E*_g_ = 3.86 eV for GZO S3. However, for a concentration over ∼6 at% Ga the band gap widening was restricted due to a decrease in carrier density on heavily-doped samples.^87^ These optical properties not only exceed standard values for FTO coatings but are commonly rare in GZO coatings while highly desired for several optical applications including low-emissivity glazing, IR imaging, light harvesting and non-linear optics.^88,89^ Carrier concentrations, mobilities and resistivities of ZnO and GZO films S1–S5 were determined by Hall effect measurements using the van der Pauw method (Fig. 6a). The doping efficiency (*η*_DE_) of films was determined as the ratio of the carrier concentration (*N*_b_) to the gallium atomic concentration in GZO films (Table 2).

**Table 2:** Carrier concentration (*N*_b_), Hall mobility (*μ*), resistivity (*ρ*), sheet resistance (*R*_sh_), doping efficiency (*η*_DE_), average transmittance at *λ* = 550 nm (*T*_*λ*550_), average transmittance in the region *λ* = 400–700 nm (*T*_*λ*(400–700)_), plasmon edge (*λ*_(plasmon)_), band gap (*E*_g_) and figure of merit (F.o.M.) for ZnO and GZO films with different at% Ga. Values for TEC™8, TEC™15, and AsahiU™ commercial standards^91,92^ are added for analogy

Sample ID	S0	S1	S2	S3	S4	S5	TEC™8	TEC™15	AsahiU™
*N*_b_ (10^20^)/cm^−3^	0.21	1.04	5.60	8.99	7.97	4.09	5.30	5.60	2.20
*μ*/cm^2^ V^−1^ s^−1^	27.4	17.8	19.8	14.7	10.0	7.4	28	21	32
*ρ* (10^−3^)/Ω cm	11.02	3.37	0.56	0.47	0.79	2.05	0.52	0.53	0.88
*R*_sh_/Ω □^−1^	186.1	64.8	9.4	9.4	14.9	36.6	8.0	15.1	9.8
*η*_DE_/%	—	50.1	90.0	65.7	37.0	15.9	—	—	—
*T*_*λ*550_/%	75	88	81	82	84	77	83	85	83
*T*_*λ*(400–700)_/%	73	85	78	80	79	75	81	83	—
*λ*_(plasmon)_/nm	—	—	1566	1292	1289	1578	1644	1751	—
*E*_g_/eV	3.32	3.47	3.59	3.86	3.84	3.76	3.91	3.97	—
F.o.M./Ω^−1^	0.4	1.4	8.6	8.7	5.6	2.1	10.4	5.7	8.5

Values were calculated considering that every incorporated gallium atoms provides one free electron with substitution of a Zn^2+^ ion, and following eqn (1), where *N*_b_ is the electron concentration, *d* is the film density (assumed as the density of ZnO, 5.606 g cm^−3^), *N*_A_ is the Avogadro constant, *c* is the gallium atomic ratio and *M* the molecular weight of ZnO.1
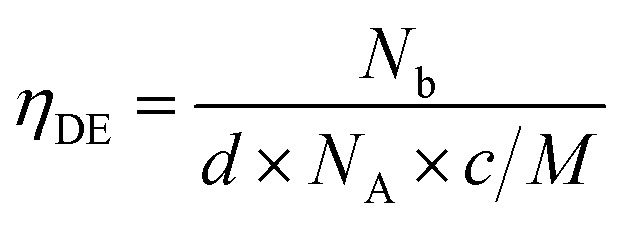


All the films behaved as n-type semiconductors, and substitution of Zn^2+^ ions with Ga^3+^ in doped samples generated free electron carriers until the dopant “saturation limit” was reached, while further addition made the excess inactive gallium atoms to act as electron scattering centres and therefore increasing sample resistivities. While ZnO exhibited the lowest carrier concentration (*N*_b_ = 2.1 × 10^19^ cm^−3^), addition of gallium atoms quickly boosted the carrier density to ∼10^20^ cm^−3^, and a strong relationship between carrier concentration and band gap enhancement of GZO can be detected (Fig. S6†). The band gap increase per unit carrier concentration shrunk as the carrier concentration increased, manifested as a deviation from the linear *E*_g_*vs. N*_b_^2/3^ plot assumed by a parabolic conduction band in the Moss–Burstein model, from which the electronic band gap in the absence of band filling effects is predicted to be 3.17 eV (Fig. S7†). Using the Moss–Burstein relation, the calculated electron mass is m* ∼ 0.25*m*_0_ at low carrier concentrations. However, as doping increased, m* appeared to increase dramatically to an unreasonable value over 1*m*_0_, (Fig. S8†) which implies a deviation from linearity due not just to conduction band non-parabolicity but also to renormalization effects. Thus, although the optical band gap widened with increasing doping concentration, the electronic band gap shrank (Fig. 6b and c). This effect occurs due to electron-dopant and electron–electron correlation effects at high dopant/electron densities respectively, which increase the ionization potential of the material.^90^ Pure ZnO showed expected high Hall mobility (27.4 cm^2^ V^−1^ s^−1^), and the addition of gallium atoms to its structure promoted a gradual increased number of impurities and therefore increased electron scattering. The added sources of scattering derived from increased grain boundaries in smaller grain sizes would explain the drastic decrease of mobility for samples S4 and S5.^77^ The lifetime of scattering electrons is expected to increase with the reduction of grain boundaries in large particle sizes,^86^ which would explain the somewhat out-of-trend higher mobility of 19.8 cm^2^ V^−1^ s^−1^ detected for S2 (Table 2). In fact, the overall charge carrier and mobility values reported herein represent some of the highest values obtained for GZO thin films deposited *via* AACVD, which are typically in the range of *N*_b_ = 8 × 10^19^ to 4 × 10^20^ cm^−3^ and *μ* = 0.1–10 cm^2^ V^−1^ s^−1^.^25,29,42–44^

To evaluate overall film optoelectronic quality, figure-of-merit (F.o.M) values were calculated using the Haacke equation (eqn (2)),^93^ and a maximum value 8.7 Ω^−1^ (Table 2) confirmed best performance for GZO S3 (3.8 at% Ga) thin film as a TCO material.2
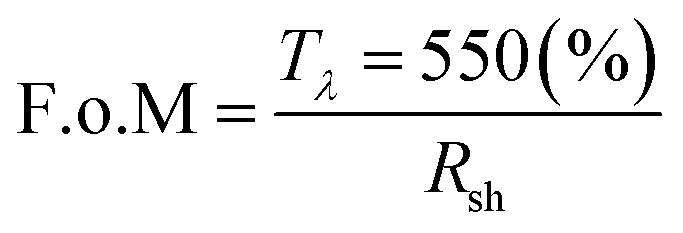


## Experimental

### Synthesis of precursor [EtZnO^i^Pr] (**1**)

The precursor synthesis was carried out by mixing a solution of ZnEt_2_ (0.1 mol, 15 wt% 1.1 M in anhydrous toluene) with anhydrous 2-propanol (0.1 mol) in anhydrous hexane (5 mL) at −78 °C (Scheme 1). Upon mixing of reagents, the solution turned cloudy and was left stirring under argon for 5 min. The reaction was then allowed to warm up to room temperature and the solution became clear. After 12 hours of storage at −20 °C, colourless crystals were collected from the solution (89%), which were not suitable for SCXRD due to their extreme moisture sensitivity.

^1^H NMR (700 MHz, C_6_D_6_): *δ* = 4.00 (sept, 1H, ^1^*J*^(H,H)^ = 9 Hz), 1.53 (t, 3H, ^1^*J*^(H,H)^ = 11 Hz), 1.19 (d, 6H, ^1^*J*^(H,H)^ = 9 Hz), 0.56 ppm (q, 2H, ^1^*J*^(H,H)^ = 11 Hz) (Fig. S9†); ^13^C{^1^H} NMR (700 MHz, C_6_D_6_): *δ* = 68.8 (CH), 27.2 (CH_3_), 12.9 (2 CH_3_), 1.7 ppm (CH_2_) (solvent residual signals (C_6_D_6_) ^1^H NMR *δ* = 7.15 ppm; ^13^C{^1^H} NMR *δ* = 128.1 ppm) (Fig. S10†). FTIR: *δ* = 2960–2855 (alkane C–H stretch), 1458–1337 (alkane C–H bend), 1130 (C–C–O *asym*. stretch), 1111 (C–O), 652 (CH_3_–C–CH_3_), 610 cm^−1^ (Zn–O) (Fig. S11 and S12†). elem. anal. (C_20_H_48_O_4_Zn_4_): calc. C, 39.11; H, 7.88. Found: C, 38.96; H, 7.82.

### Thin film deposition

AACVD experiments were carried out in a horizontal bed cold-walled 17 × 6 cm tubular reactor. Various amounts of gallium acetylacetonate [Ga(acac)_3_] were added to the precursor solution for Ga/Zn molar ratios of 1 to 16 mol% dissolved in anhydrous 2-propanol (15 mL). After placing the mixture in a glass bubbler, an aerosol mist was created using a piezoelectric device and then transported to the reaction chamber by 1.8 L min^−1^ flowing nitrogen gas (99.9%, BOC). Depositions were carried out on silica coated barrier glass to prevent unwanted leaching of ions from the glass into the thin films, which was cleaned using acetone (99%), isopropanol (99%), and distilled water and dried at 160 °C for 1 h prior to use. The substrate temperature was set to 425 °C, and the deposition times varied from 10 to 15 min. Material characterization analyses were performed in uniform areas at 3 cm from the reactor inlet.

### Analytical characterisation

Reactions were carried out under an argon atmosphere using standard Schlenk techniques. ^1^H and ^13^C{^1^H} NMR spectra were recorded in C_6_D_6_ on a Bruker Advance III 700 Cryo spectrometer operating at 700.349 and 176.103 MHz, respectively. Typical respective spectral widths for proton and carbon were 13.88 and 41.66 kHz, the pulse widths were 36.00 and 12.00 μs, and pulse delays were 7.27 s and 7.95 s, respectively. The measurements were performed at room temperature in 5 mm NMR tubes, and spectra were referenced to the solvent resonances and are reported relative to Me_4_Si. FTIR measurements were performed using a Bruker Alpha-T ATR-FTIR Fourier Transform Infrared Spectrometer. TGA/DSC were carried out in a Netzsch STA 449C instrument using aluminium crucibles with the precursor packed under an argon atmosphere, which were punctured immediately before measurement. Data points were recorded in the temperature range 20–500 °C. Grazing incidence X-ray diffraction (GIXRD) measurements were performed using a Bruker-Axs D8 (Lynxeye XE) diffractometer with monochromated Cu K_α1_ radiation (1.54184 Å; 20 kV, 5 mA). The films were analysed at a grazing incident angle of 1°. Diffraction patterns were collected over 20–66° with a step size of 0.05° and a step time of 3 s per point. Le Bail fits were carried out using structure parameters from the Physical Sciences Data-Science service (PSDS), using GSAS software suit. X-ray fluorescence spectra were recorded using a Panalytical Epsilon4 multifunctional XRF analyser with integrated calibration in helium atmosphere. X-ray photoelectron spectroscopy (XPS) was performed using a Thermo Scientific K-alpha spectrometer with monochromated Al K_α_ radiation (8.3418 Å), a dual beam charge compensation system. Survey scans were collected in the range of 0–1200 eV at a pass energy of 50 eV. Sputtering treatments (2 cycles × 200 s) were carried out by Ar^+^ bombardment at 3.5 kV, with an argon partial pressure of 5 × 10^−8^ mbar (etching rates estimated 0.5 nm s^−1^). Samples were introduced directly by a fast entry lock system into the analytical chamber. High resolution peaks were used for the principal peaks of Zn (2p), O (1s), Ga (2p), and C (1s). The peaks were modelled using sensitivity factors to calculate the film composition and the area underneath these bands was an indicator of the element concentration within the region of analysis (spot size 400 μm). Peak positions were calibrated to adventitious carbon (284.8 eV) and plotted using the CasaXPS Software. The surface morphology was evaluated using a JEOL JSM 6301F (2 kV) Field Emission SEM at an accelerating voltage of 2 keV. Film thickness was measured using cross-sectional images. Electrical properties of films were studied by the van der Pauw method at room temperature using an Ecopia HMS-3000 hall measurement system. Square-cut samples (1 × 1 cm) were subjected to a 0.58 T permanent magnet and a current of 0.5 mA during the measurement. UV/vis/near-IR transmission and reflection spectra were recorded in the range of 300 to 2500 nm using a PerkinElmer Fourier Transform Lambda 950 UV-vis-NIR spectrometer. The transmission spectra background was taken against an air background. The average visible light transmittance (380 nm to 780 nm) of the studied glasses was evaluated according to the British Standard EN 673.

## Conclusions

**Fig. 7:**
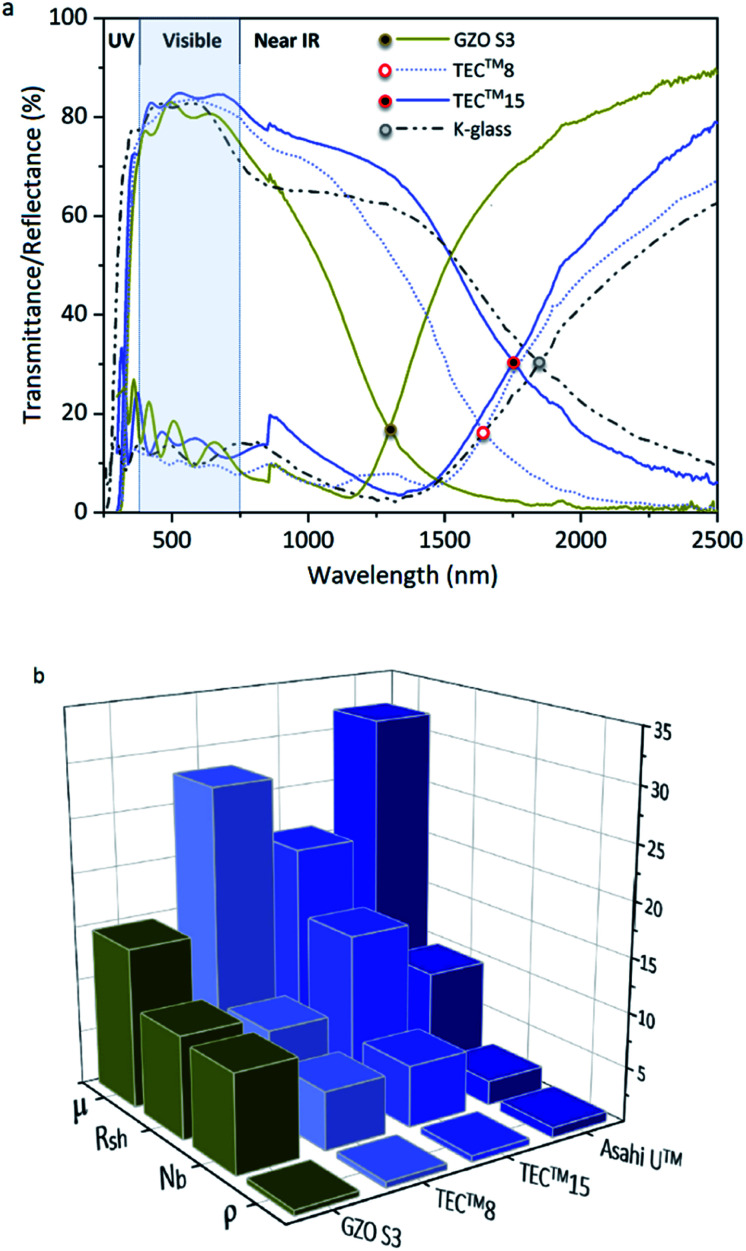
(a) Optical properties of GZO S3 thin films and standards TEC™8, TEC™15 (ref. 91 and 92) and low-E coating K-Glass®;^94^ (b) Electronic properties of GZO S3 thin films and standards TEC™8, TEC™15 and Asahi U™:^91,92^ Resistivity (*ρ* (×10^−3^)/Ω cm), charge carrier concentration (*N*_b_ (x10^20^)/cm^−3^), sheet resistance (*R*_sh_/Ω □^−1^) and mobility (*μ*/cm^2^ V^−1^ s^−1^).

The optoelectronic values achieved in GZO thin films reported herein are comparable to those of highly conductive GZO deposited through high-vacuum methods,^24,33,34,74^ with resistivity values 10–100 times lower than previously reported for GZO films synthesized *via* AACVD.^25,29,42–44^ Highly (002)-textured and compact GZO thin films were successfully deposited on glass substrate *via* AACVD from a non-halogenated/non-corrosive pre-organized zinc precursor [EtZnO^i^Pr]_4_ and [Ga(acac)_3_] using a single-inlet deposition method. The optimal doping limit or “saturation limit” of the materials was confirmed to be ∼3–4 at% Ga, in which the most acute *c*-axis growth is promoted alongside a morphology of large compact flat/shallow particles. Irregular granular agglomerated particles appear for lower and higher gallium concentrations, resulting from increasing growth of non-(002) surfaces. GZO films exhibit high visible transmittance (77–88%), good infrared reflection (>50% at 1500 nm and 80–90% at 2500 nm) and plasma resonance wavelengths in the range of 1290–1560 nm. High carrier density (∼10^20^ to 10^21^ cm^−3^) and reasonable mobility values (10–20 cm^2^ V^−1^ s^−1^) are observed for lightly doped thin films (∼1–6 at% Ga). GZO S3 (3.8 at% Ga) films exhibit the best combined overall optoelectronic properties: maximum carrier concentration (*N*_b(max)_ = 8.99 × 10^20^ cm^−3^), minimum resistivity (*ρ*_(min)_ = 4 × 10^−4^ Ω cm; *R*_sh_ = 9.4 Ω·□^−1^, thickness ∼600 nm), high transparency (>80%), maximum band gap enhancement (*E*_g(max)_ = 3.86 eV) and minimum plasmon resonance wavelength (*λ*_(plasmon)min_ = 1250 nm, *R*_%1300 nm_ > 85%). Maximum degree of self-texturing was also found for this sample (TC(002)_max_ = 4.8), as well as the most uniform and smooth cross-sectional and surface morphology. In conclusion, GZO S3 thin film properties make this coating an outstanding, sustainable and cheap alternative to commercial coatings currently used in the photovoltaic industry and as a low-emissivity coating for energy glazing, readily comparable to commercial standards (Fig. 7). The use of a ZnO molecular precursor with pre-formed Zn–O cube-like core and zinc-oxygen ratio restricted to (1 : 1) in the metal 1^st^ coordination sphere that can undergo a thermal polymerization-type decomposition path has proved to be of key importance in the generation of highly oriented and dense GZO thin films. Additionally, the use of low-cost reagents, a fast and halogen-free synthetic route with easy elimination of decomposition by-products to generate highly pure and durable crystalline films with excellent optical and electrical properties showcases the outstanding potential that AACVD can bring to industrial manufacturing of TCOs. The cost-effective approach to energy-efficient GZO coatings herein reported – through combination of Schlenk chemistry and AACVD – can generate highly crystalline films with optical properties that exceed standard values for commercial low-E coatings and solar applications. The use of pre-organized precursors that can form highly (002)-textured and oxygen-deficient ZnO films together with the morphological versatility that can be achieved using solution-based methods described herein pushes GZO thin films one step closer to be a solid candidate to replace the expensive and halogenated TCOs that currently dominate the market for technical applications.

## Conflicts of interest

There are no conflicts to declare.

## Supplementary Material

SC-011-D0SC00502A-s001

SC-011-D0SC00502A-s002

SC-011-D0SC00502A-s003

SC-011-D0SC00502A-s004

SC-011-D0SC00502A-s005

SC-011-D0SC00502A-s006

SC-011-D0SC00502A-s007

SC-011-D0SC00502A-s008

SC-011-D0SC00502A-s009

SC-011-D0SC00502A-s010

SC-011-D0SC00502A-s011

SC-011-D0SC00502A-s012

SC-011-D0SC00502A-s013

SC-011-D0SC00502A-s014
