# Supplementary figures and images for: Aerosol-assisted route to low-E transparent conductive gallium-doped zinc oxide coatings from pre-organized and halogen-free precursor
Source: Chem Sci. 2020 Apr 27;11(19):4980–90. doi: 10.1039/d0sc00502a (PMC8159247; doi:10.1039/d0sc00502a)

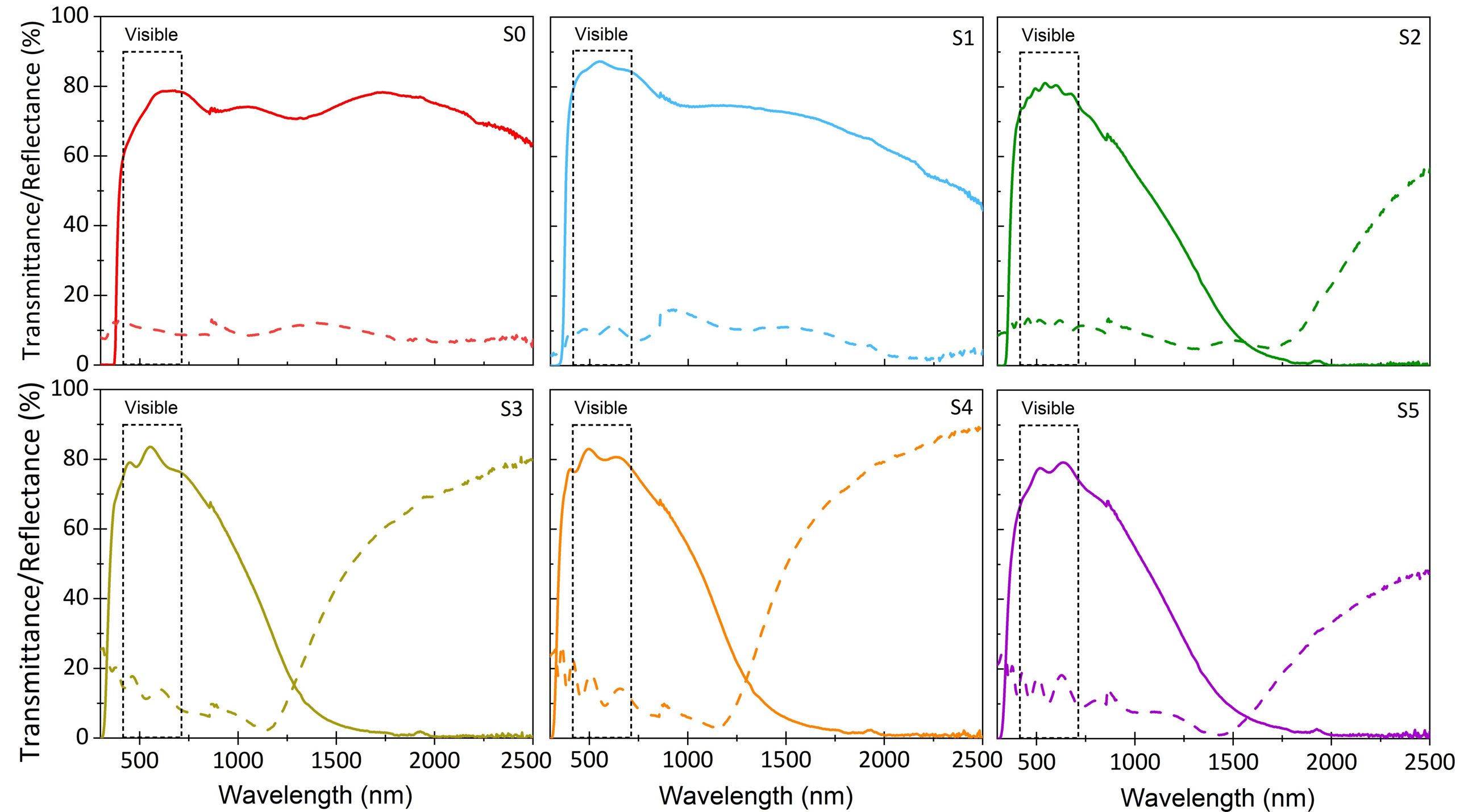

Supplement: SC-011-D0SC00502A-s002 [file SC-011-D0SC00502A-s002.pdf]

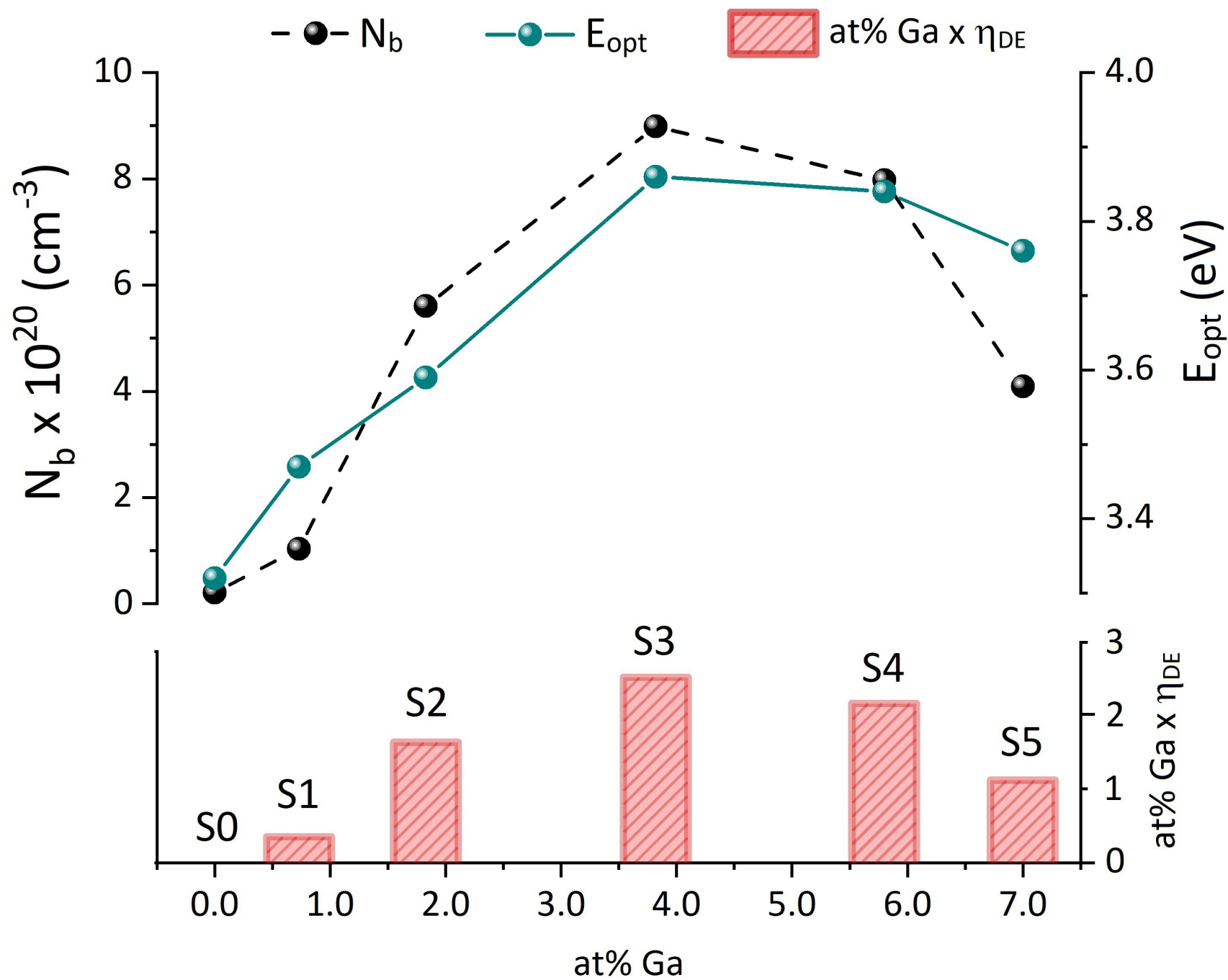

Supplement: SC-011-D0SC00502A-s003 [file SC-011-D0SC00502A-s003.pdf]

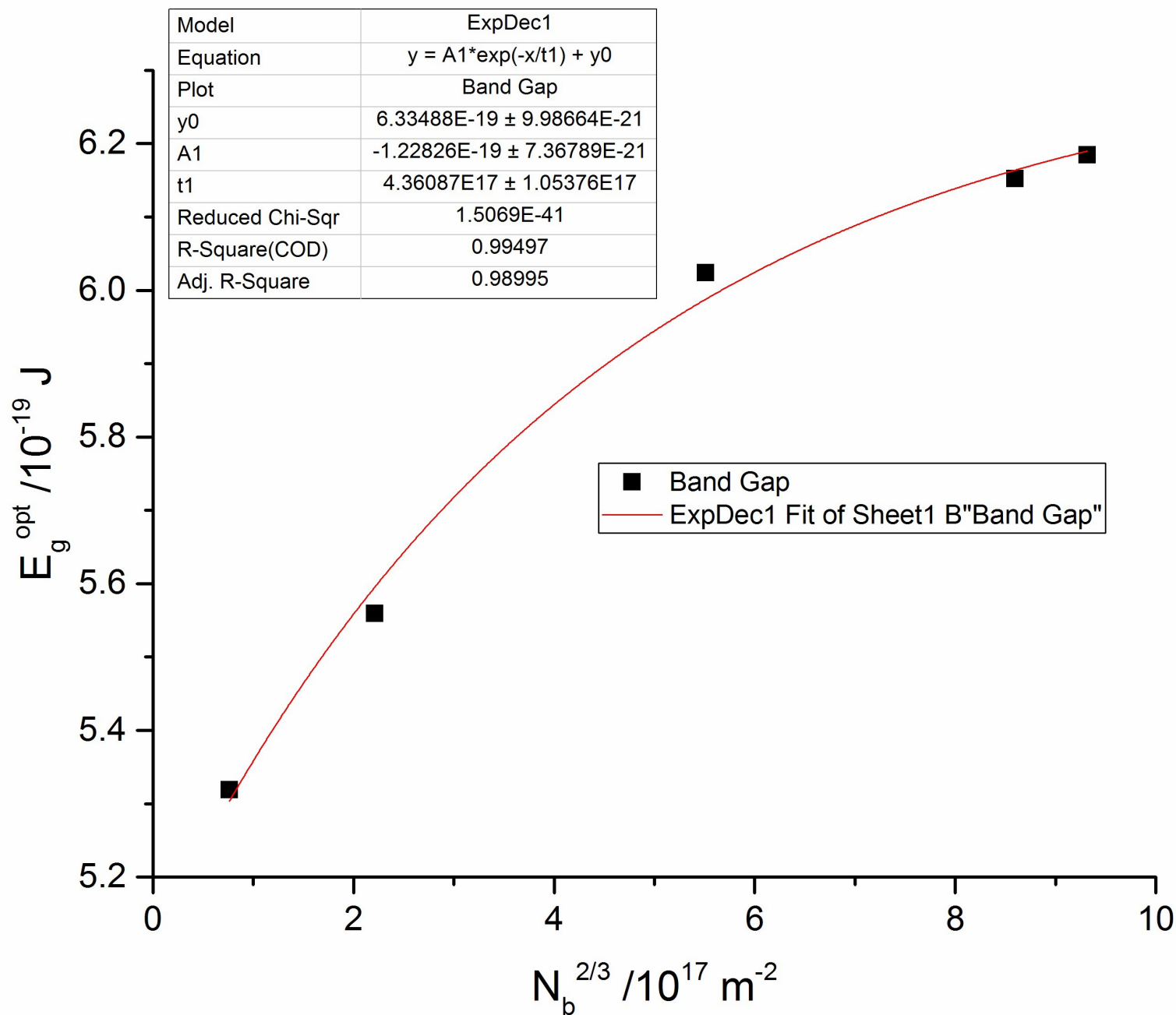

Supplement: SC-011-D0SC00502A-s004 [file SC-011-D0SC00502A-s004.pdf]

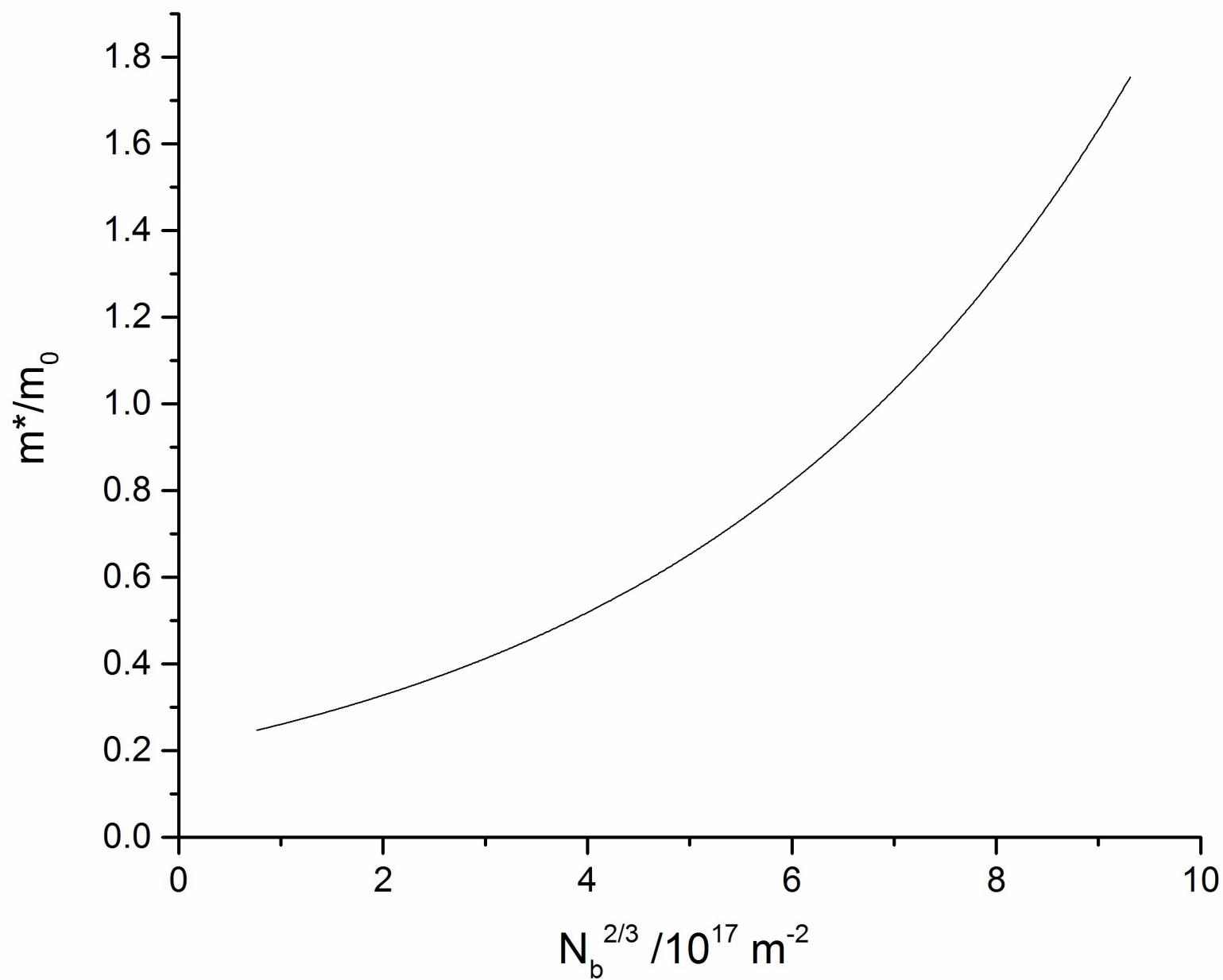

Supplement: SC-011-D0SC00502A-s005 [file SC-011-D0SC00502A-s005.pdf]

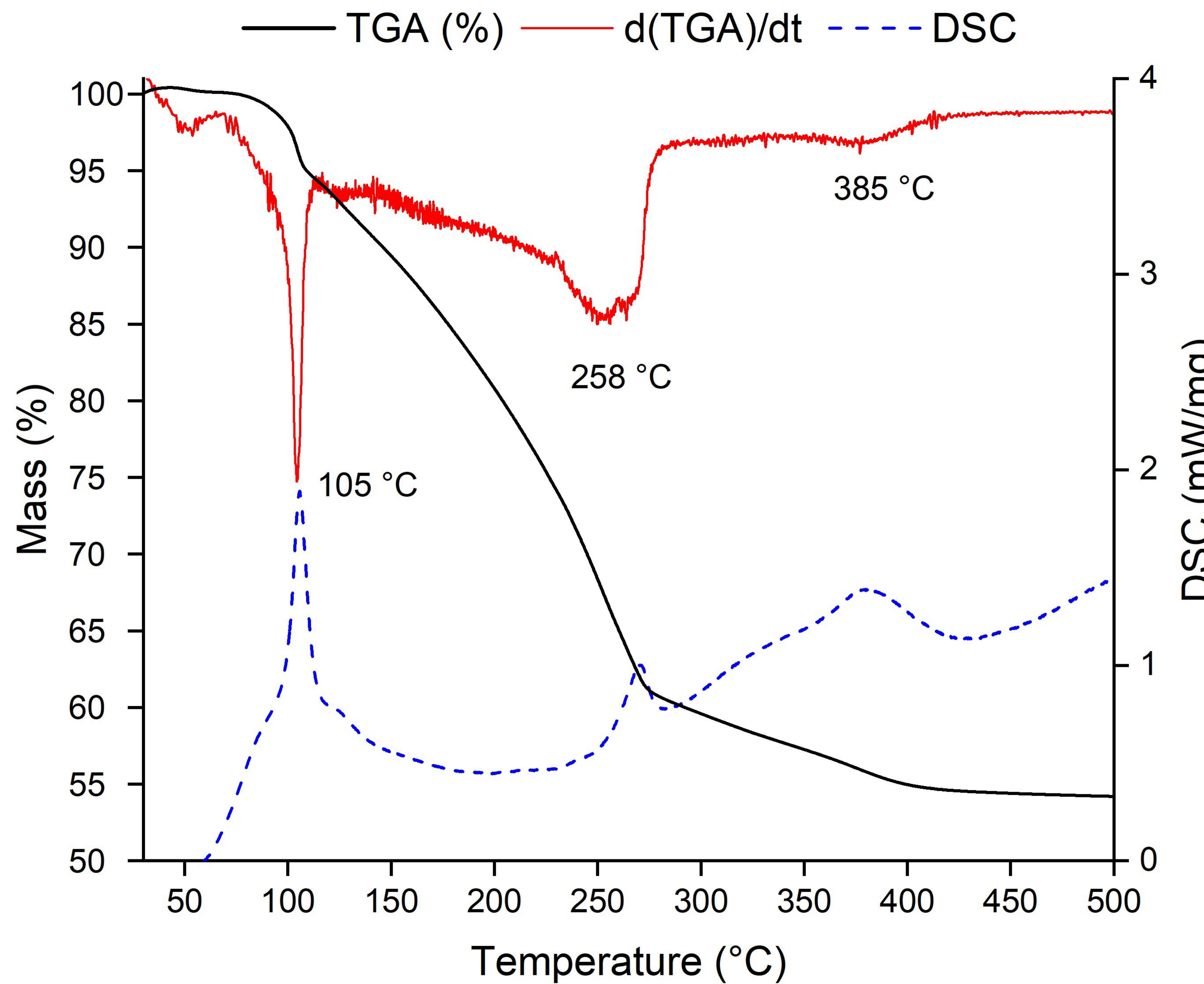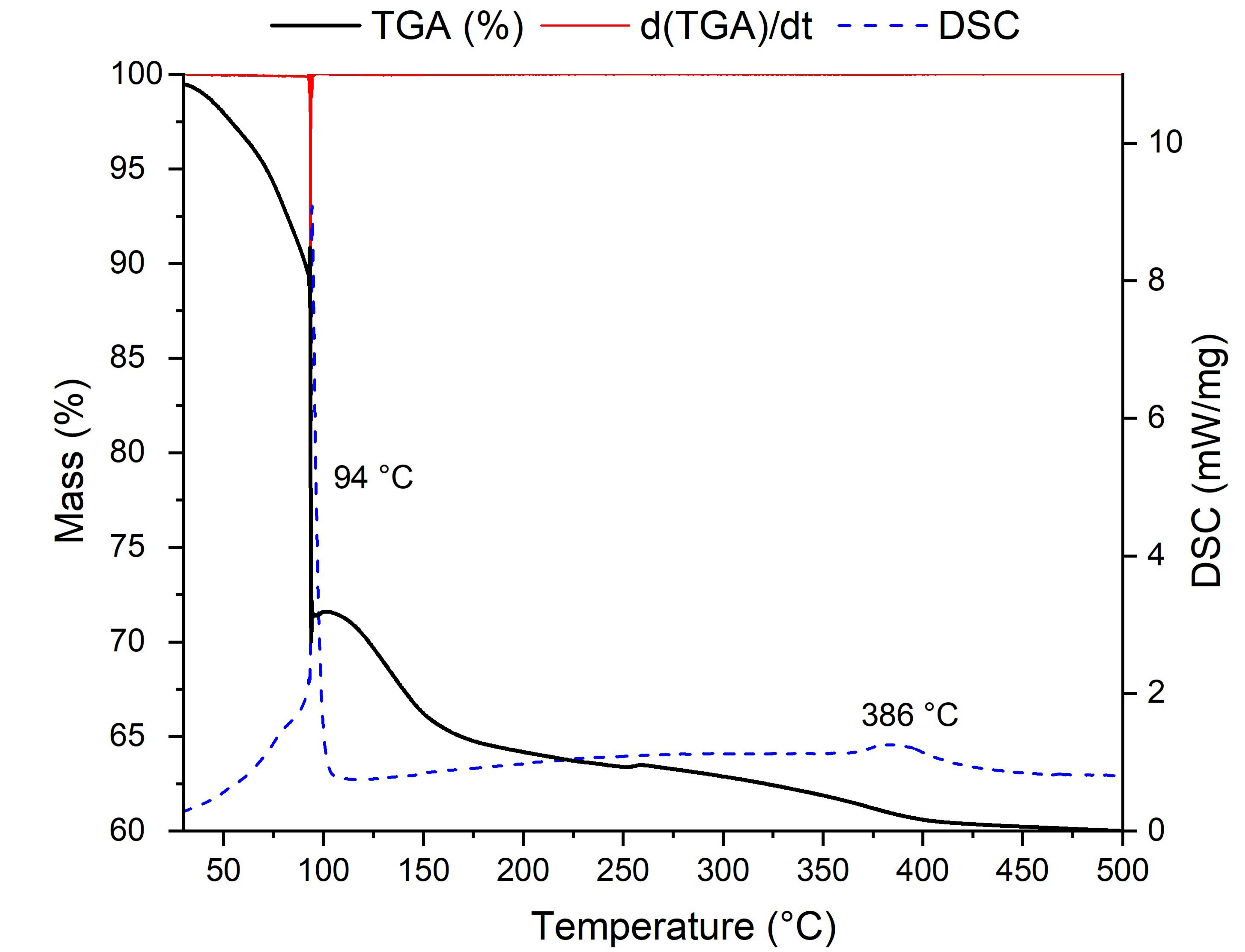

Supplement: SC-011-D0SC00502A-s008 [file SC-011-D0SC00502A-s008.pdf]

$^{13}\text{C}$  NMR ("EtZnOiPr")<sub>n</sub>

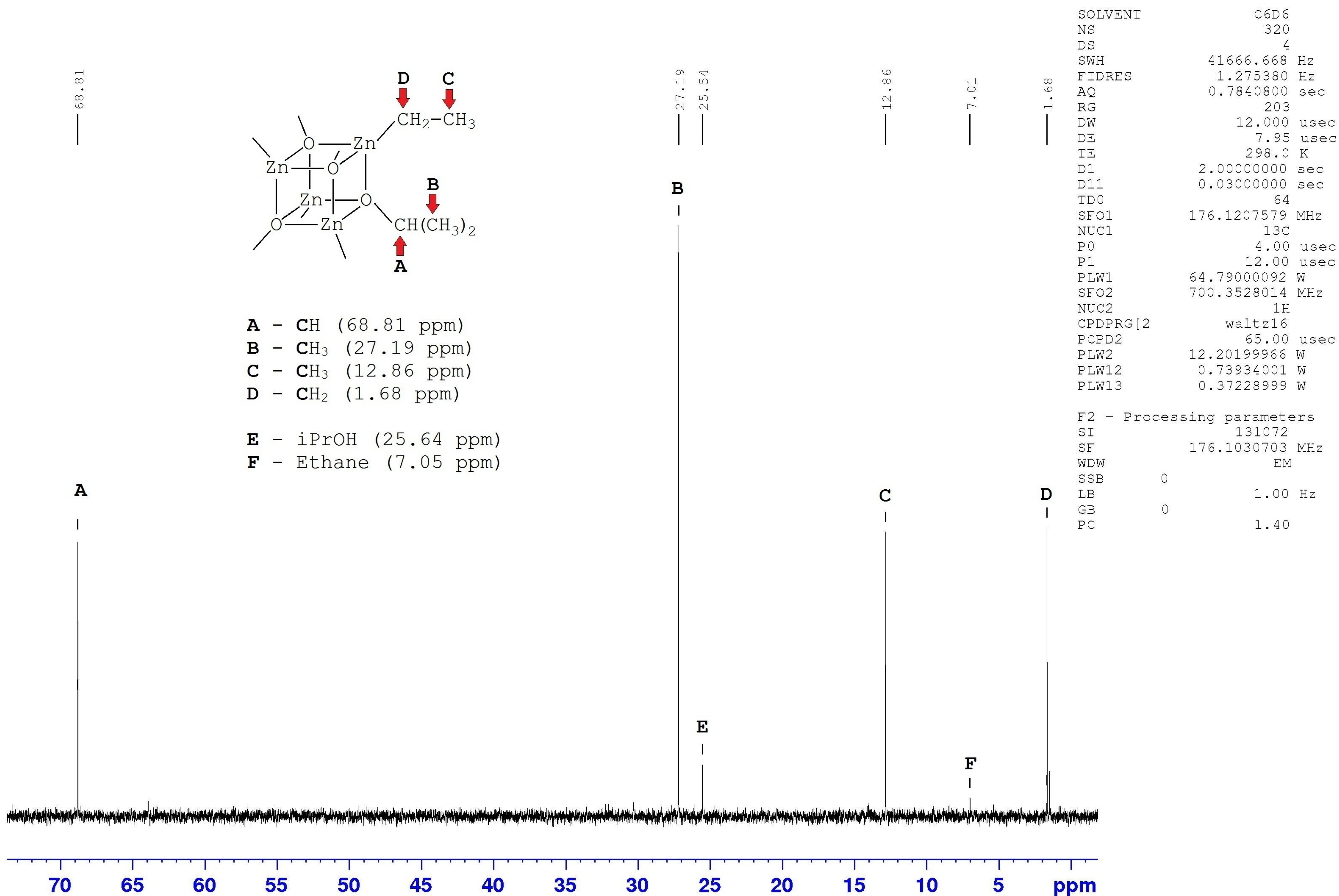

Supplement: SC-011-D0SC00502A-s009 [file SC-011-D0SC00502A-s009.pdf]

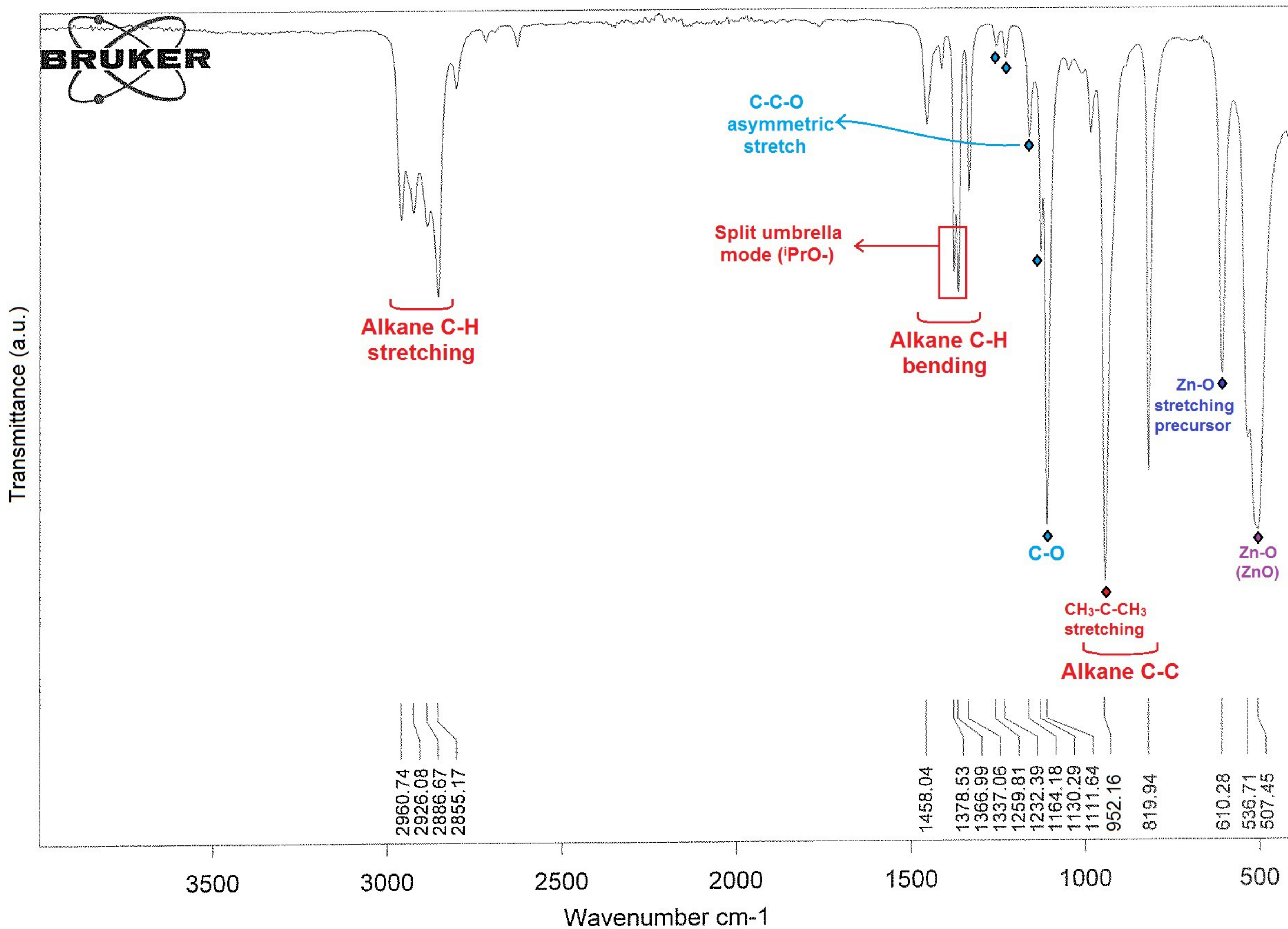

Supplement: SC-011-D0SC00502A-s010 [file SC-011-D0SC00502A-s010.pdf]

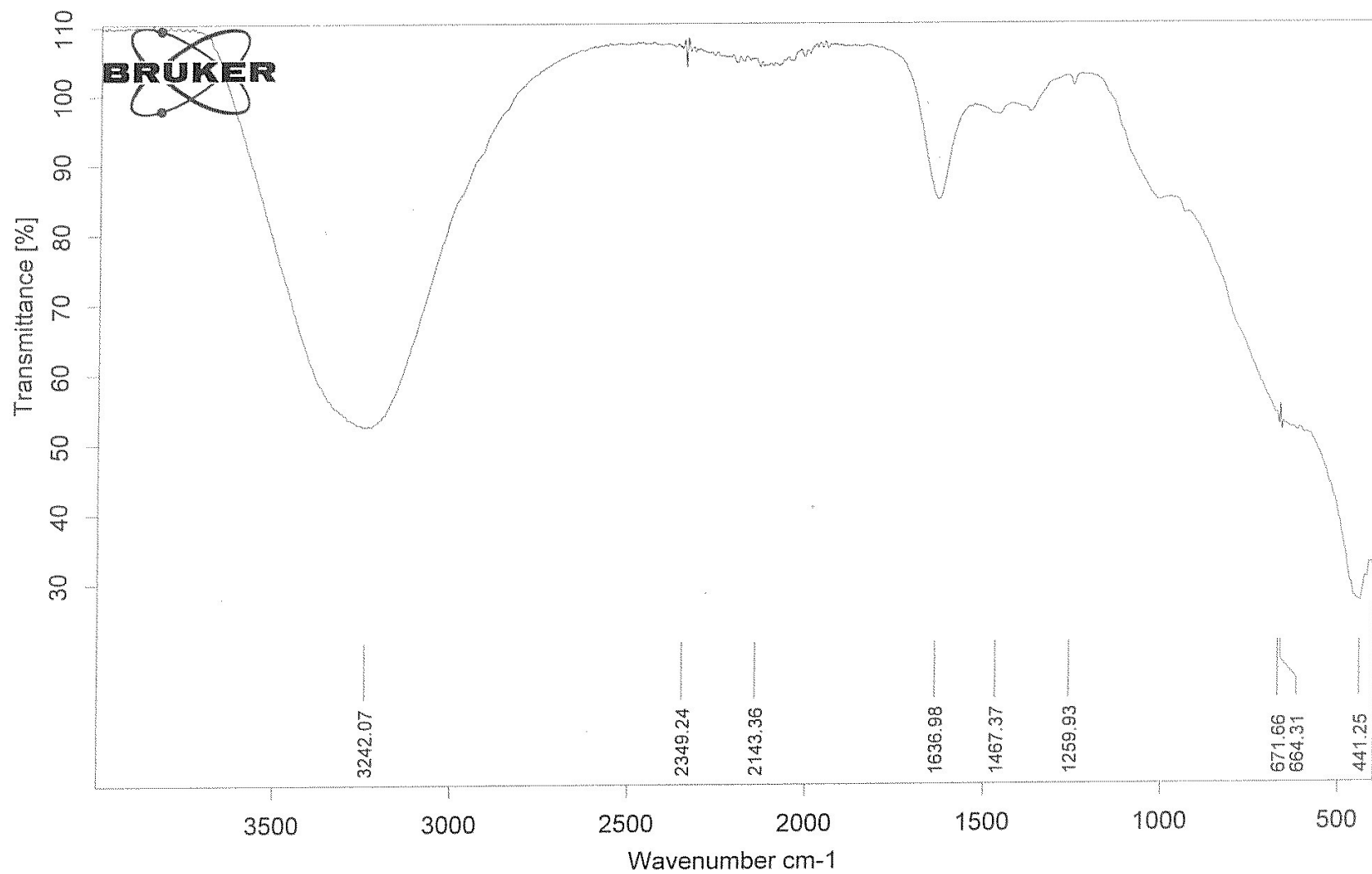

Supplement: SC-011-D0SC00502A-s011 [file SC-011-D0SC00502A-s011.pdf]

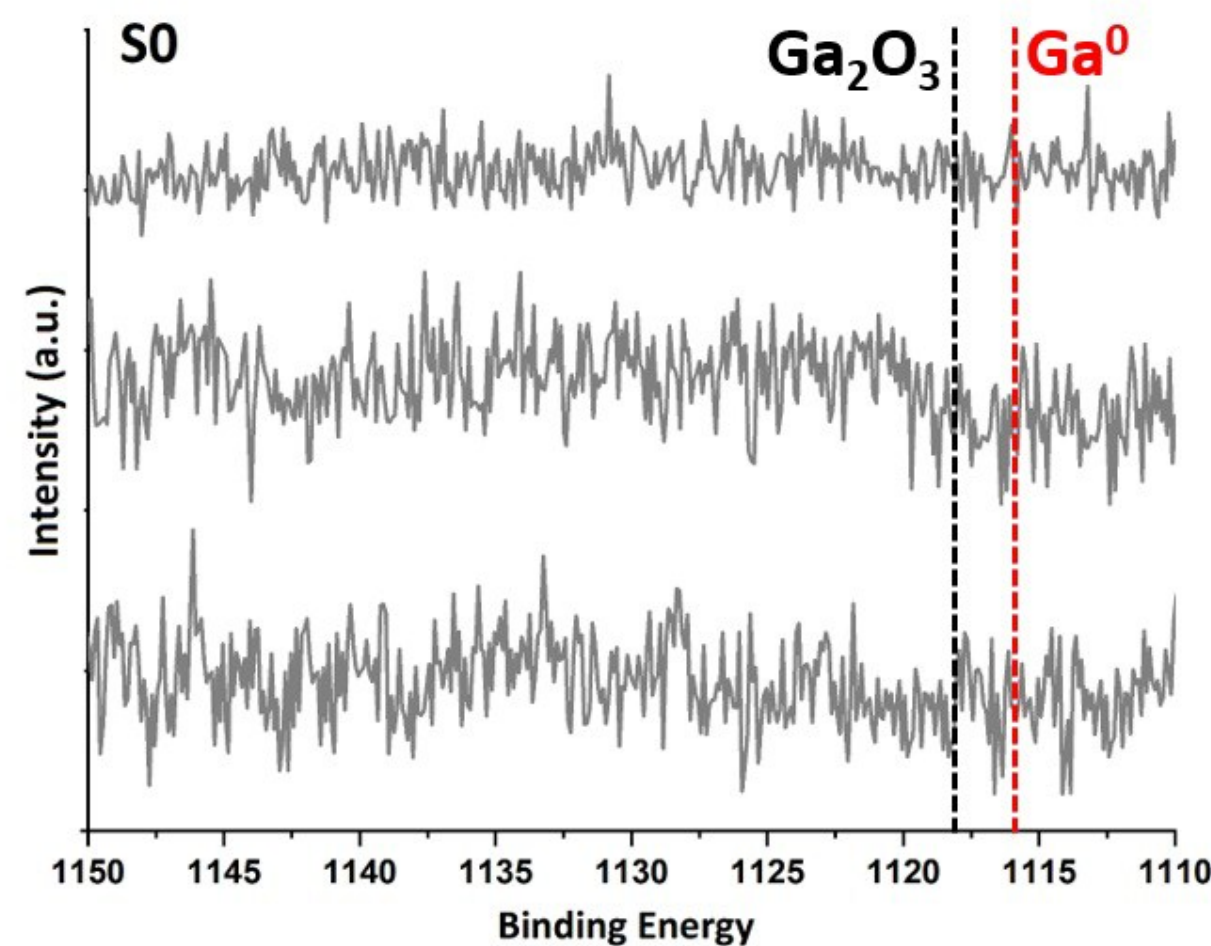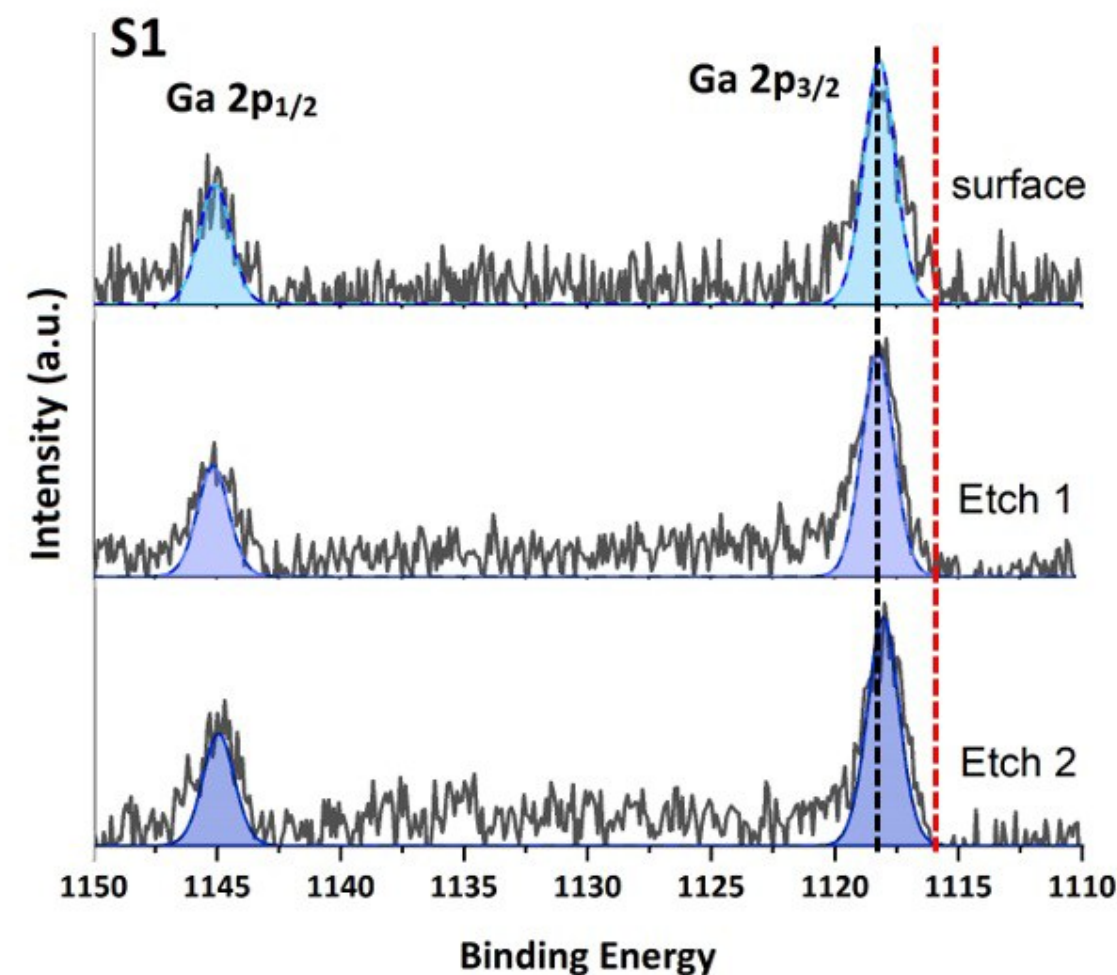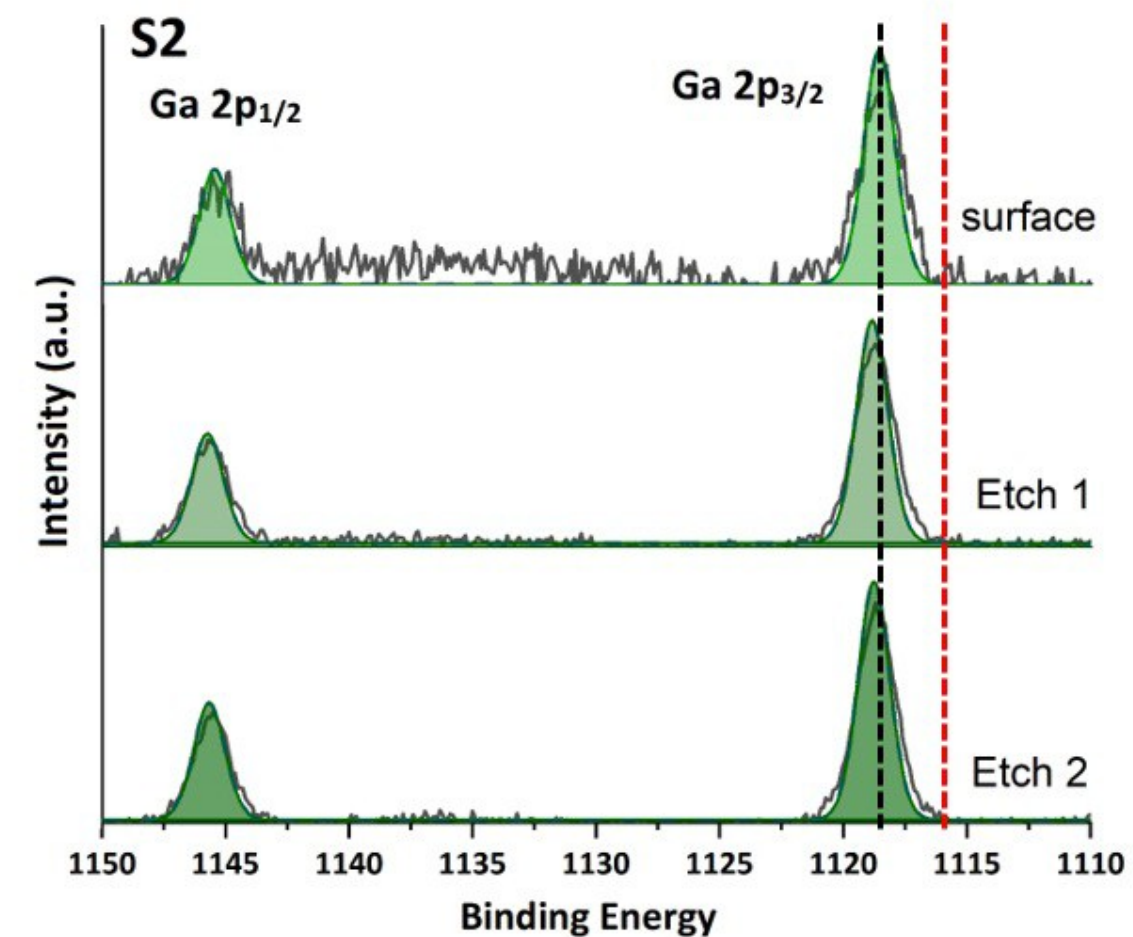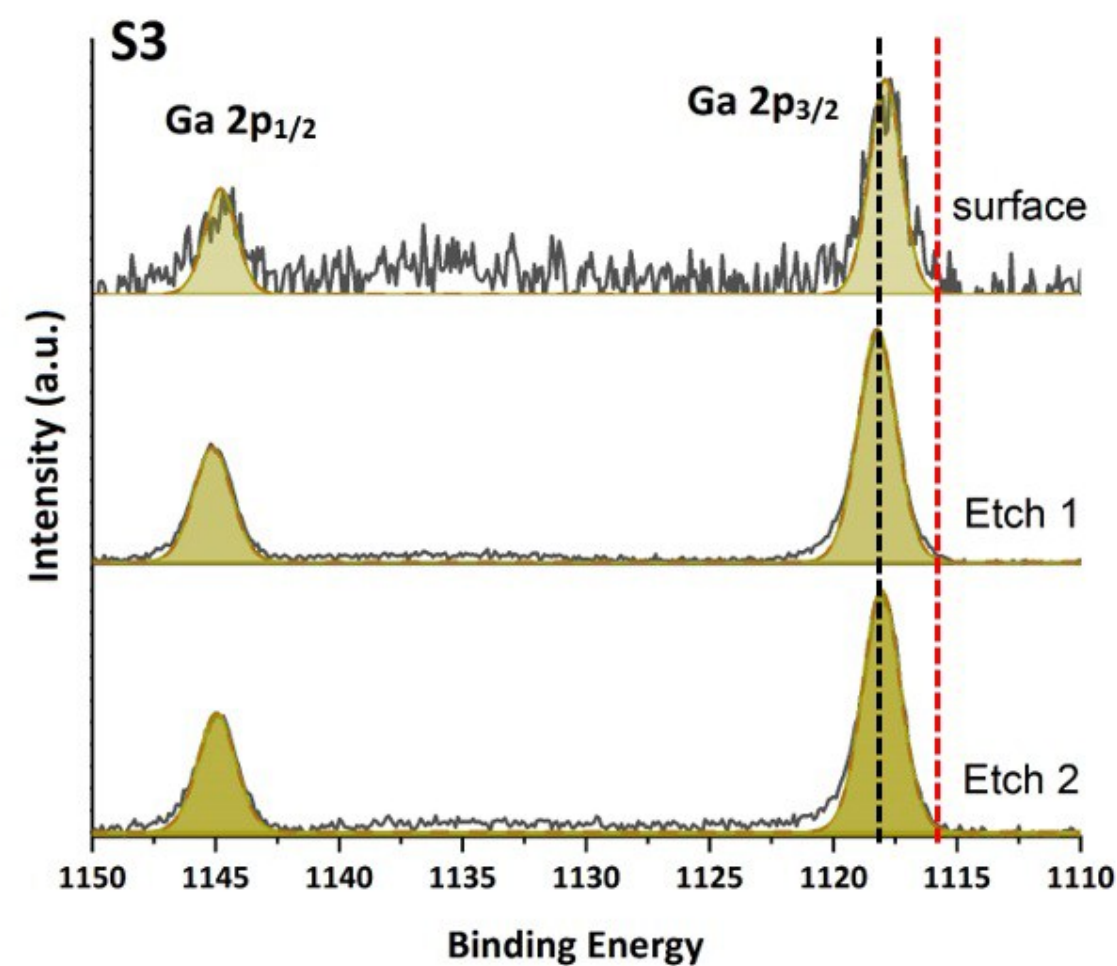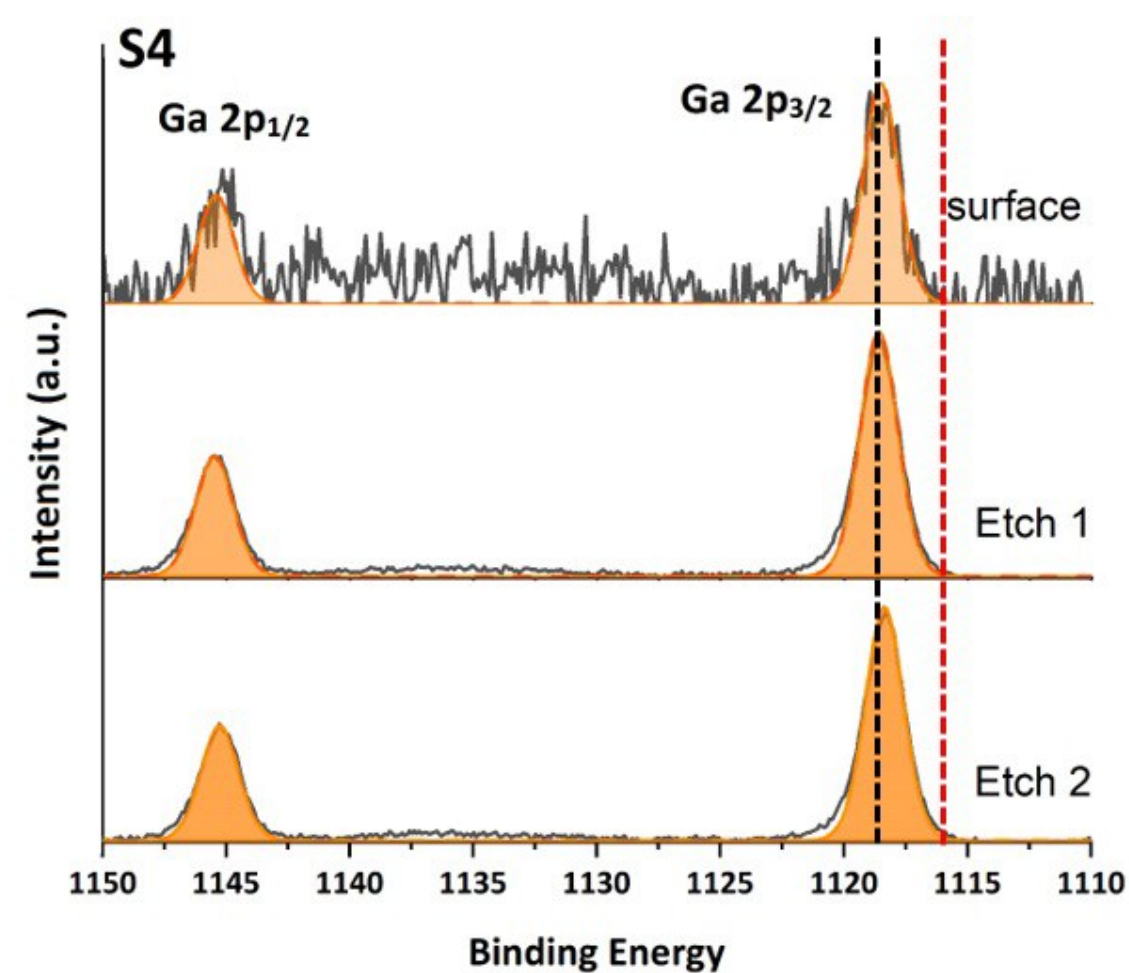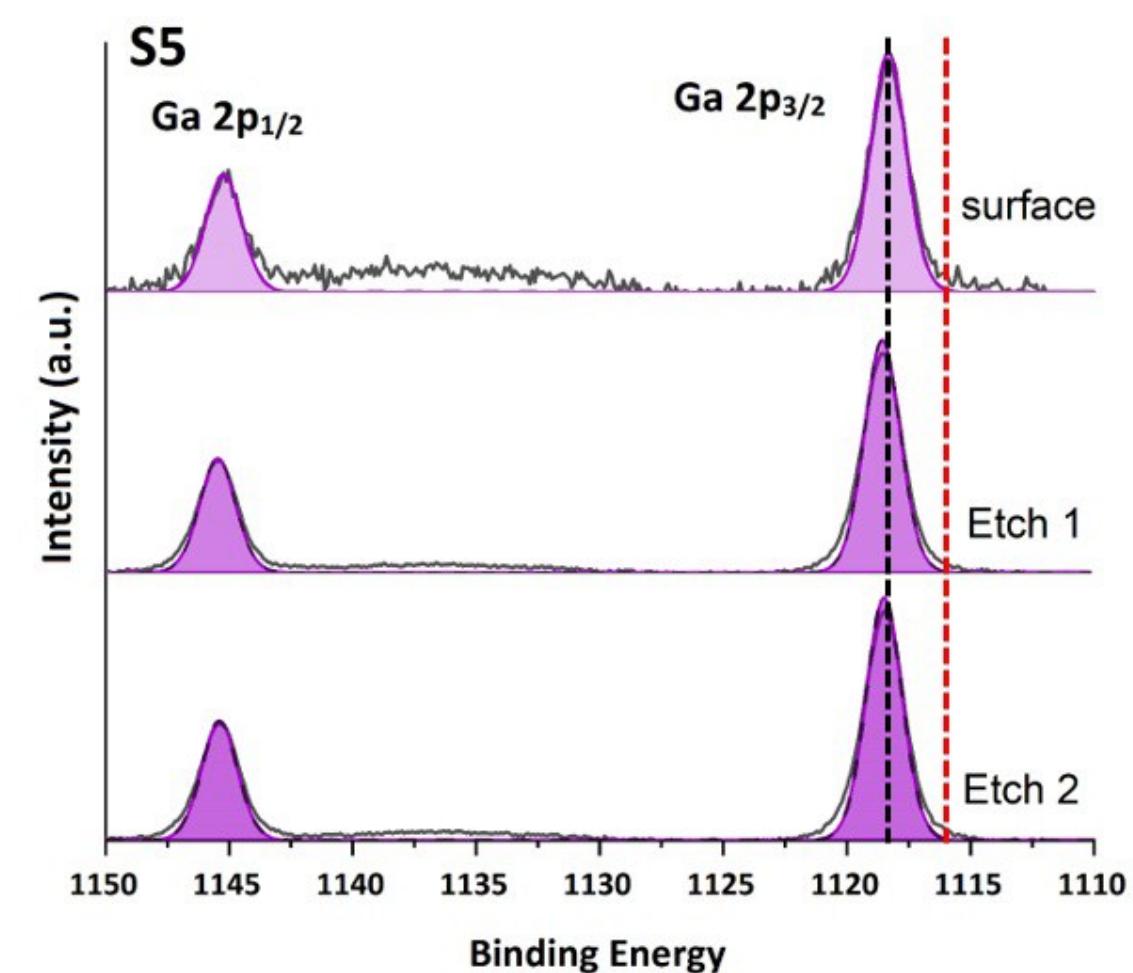

Supplement: SC-011-D0SC00502A-s012 [file SC-011-D0SC00502A-s012.pdf]

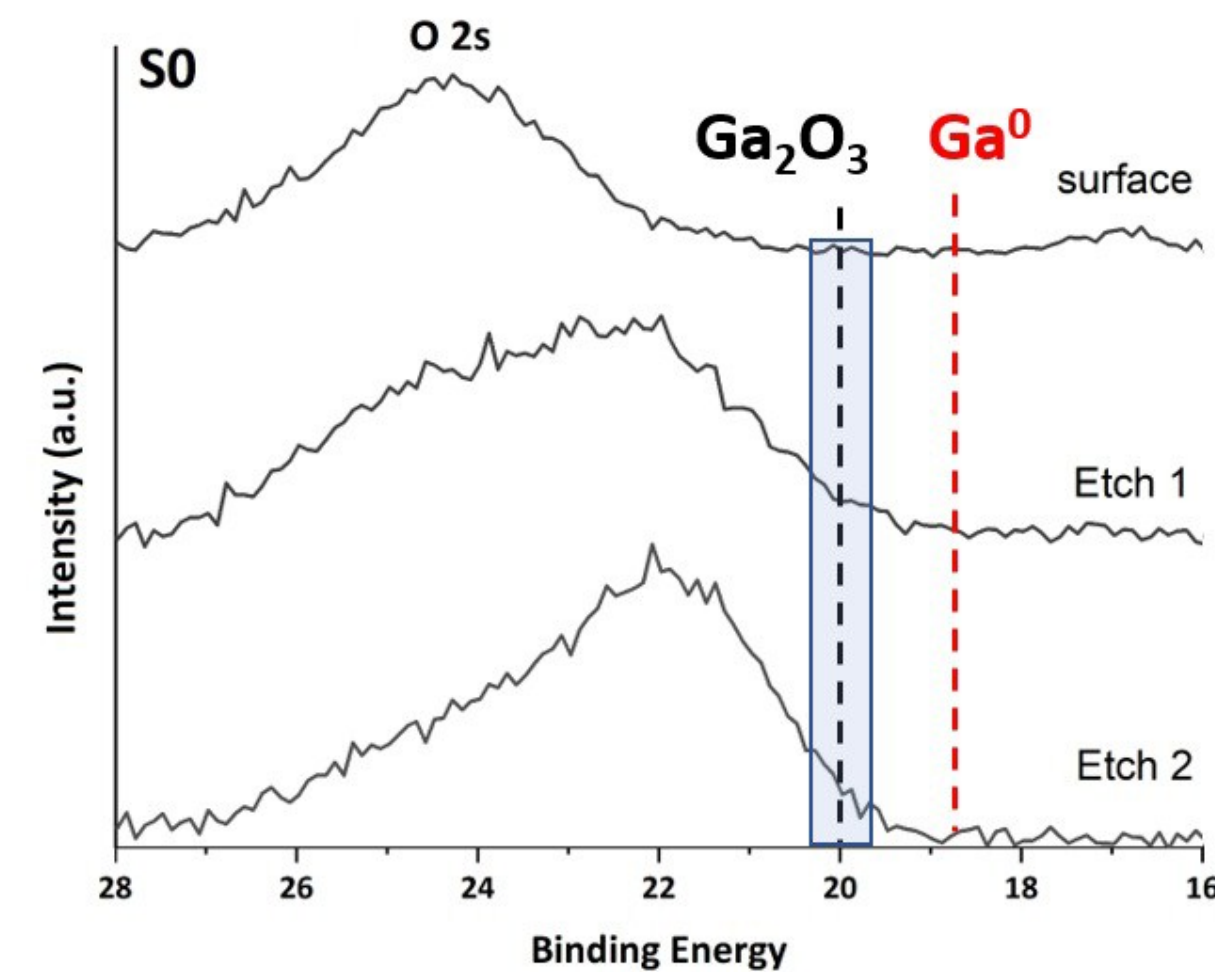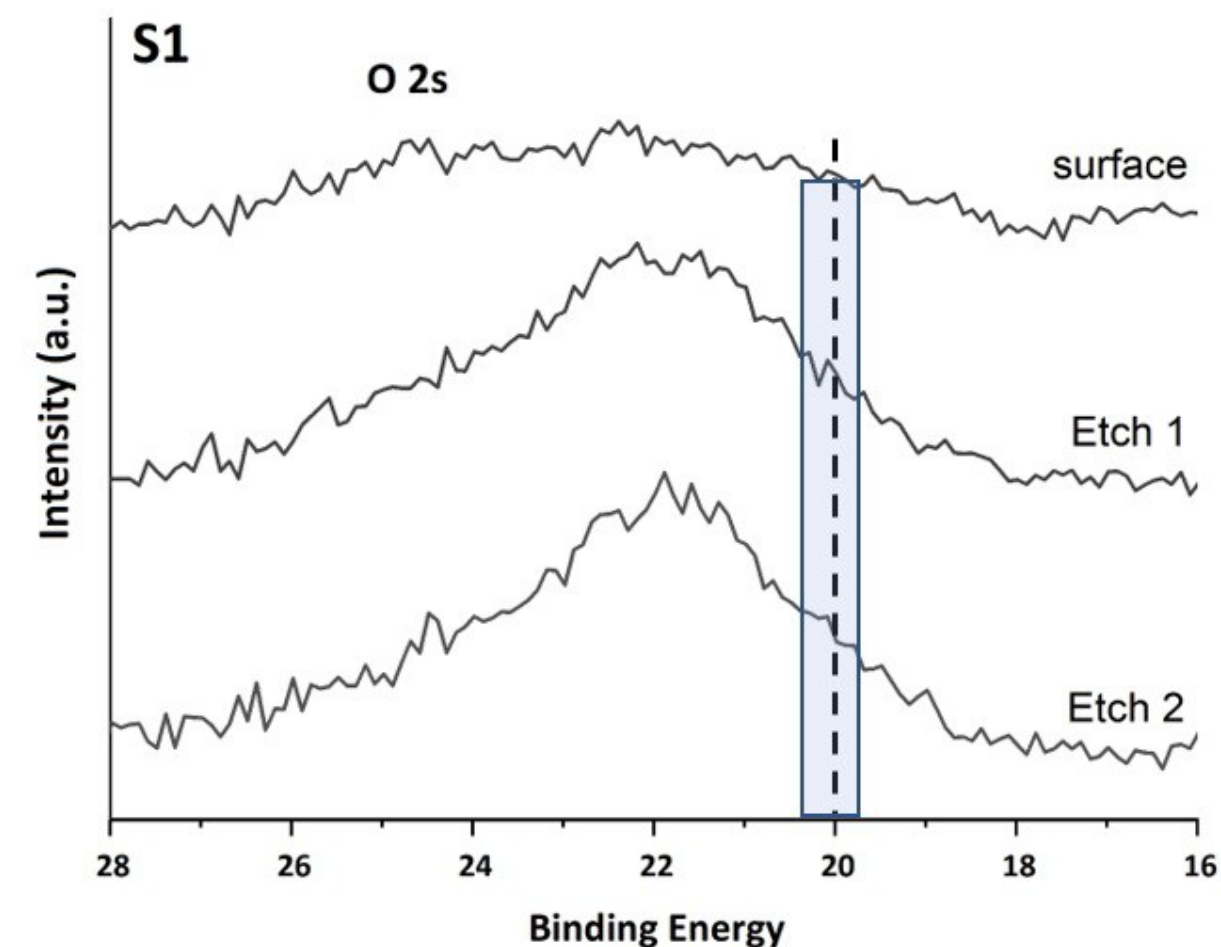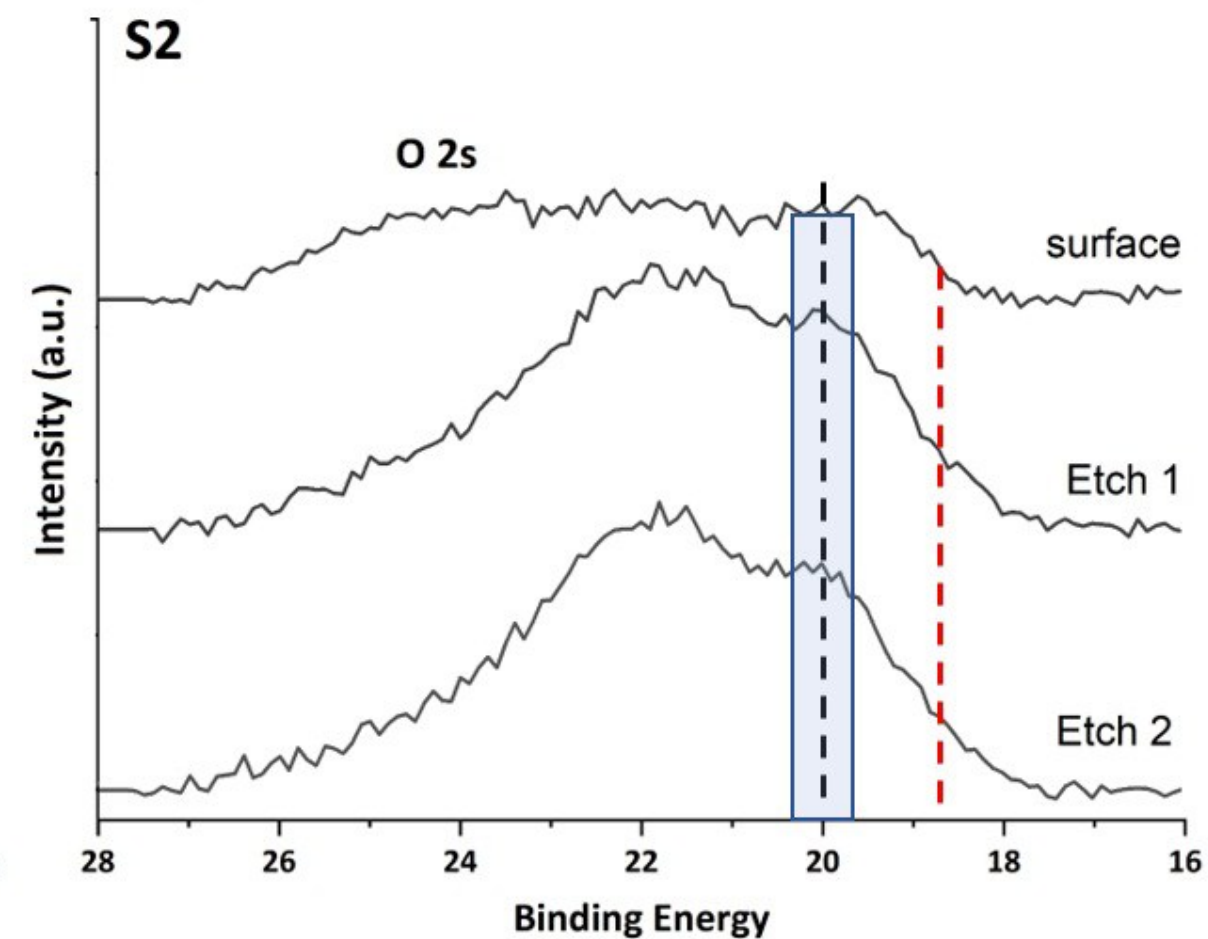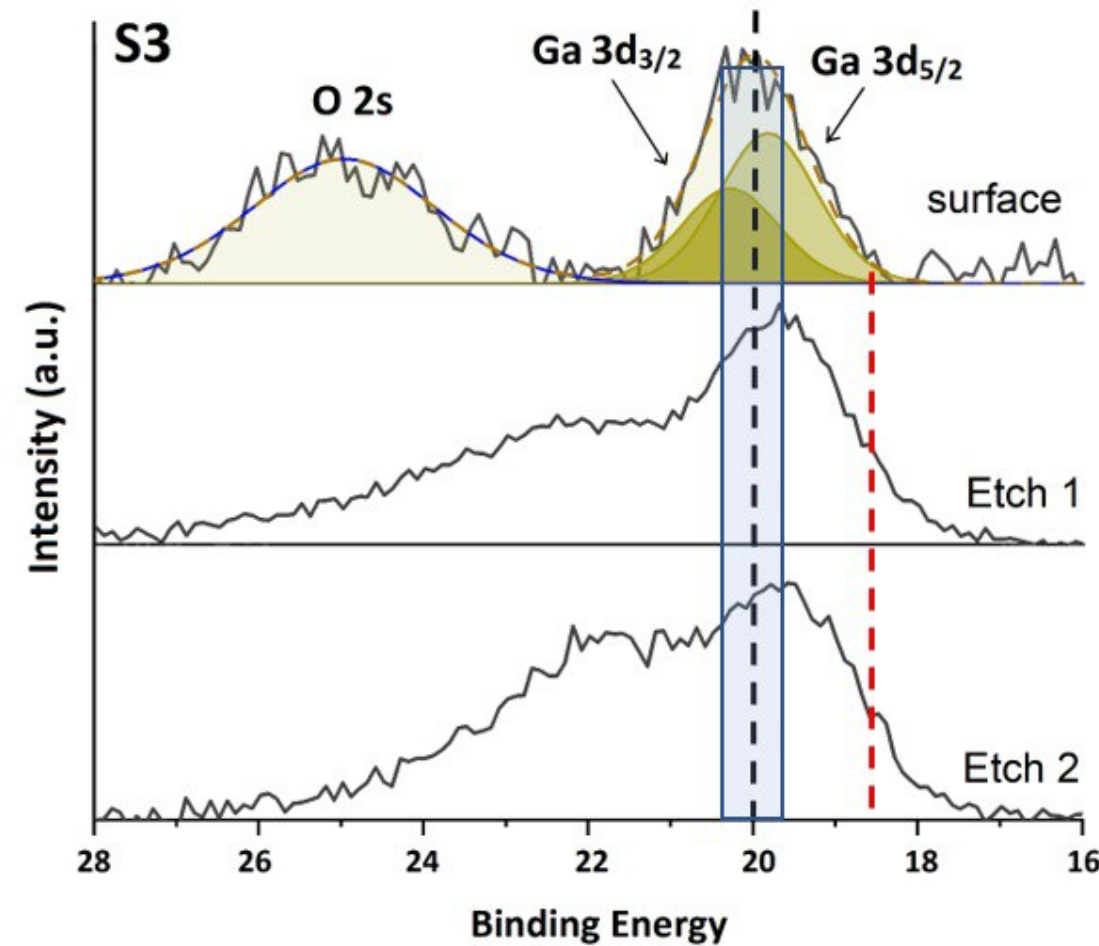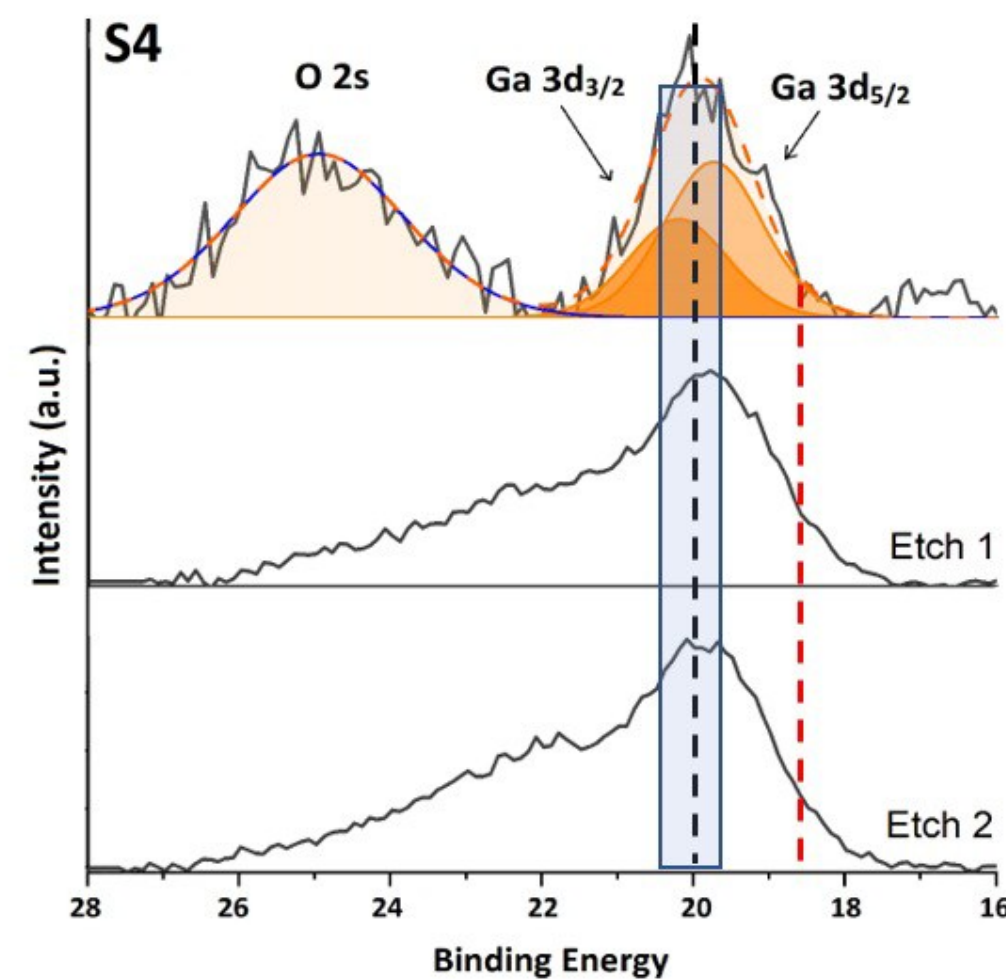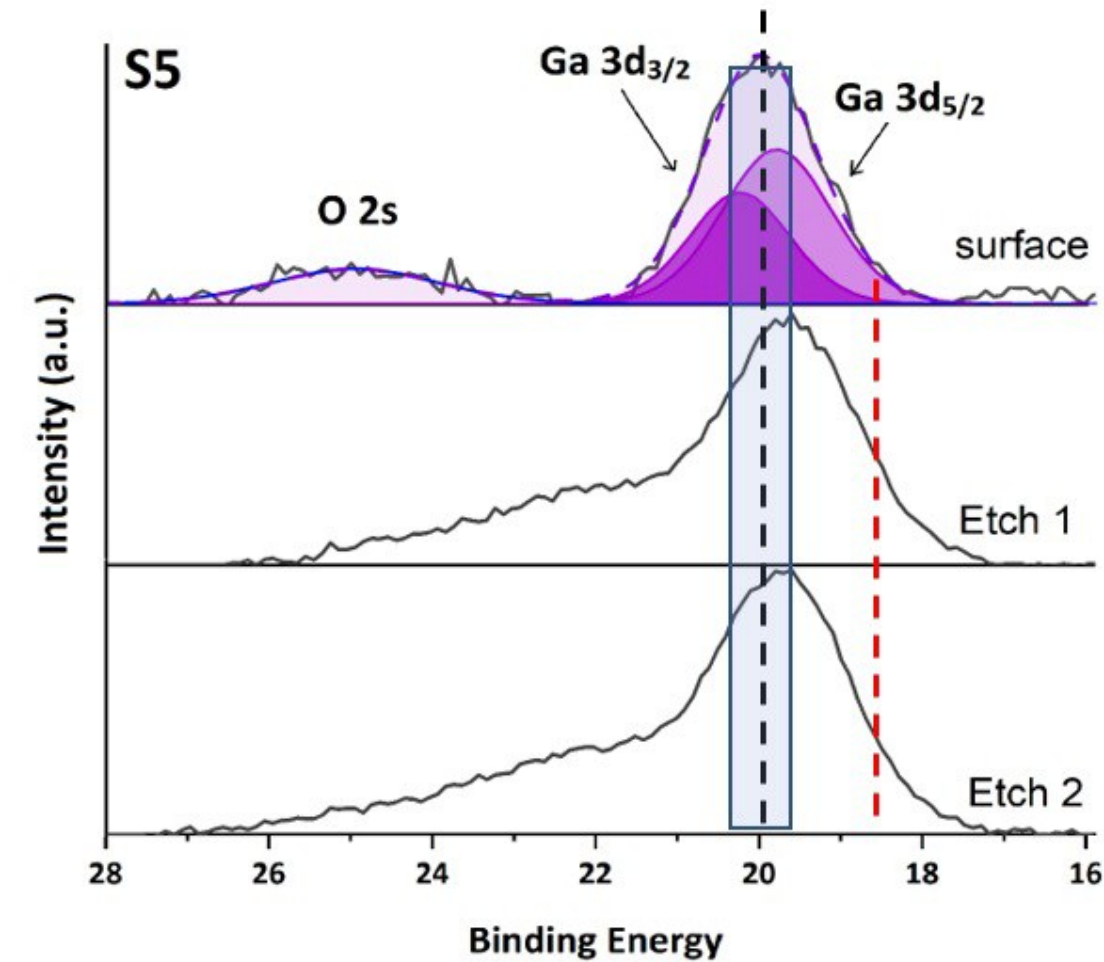

Supplement: SC-011-D0SC00502A-s013 [file SC-011-D0SC00502A-s013.pdf]

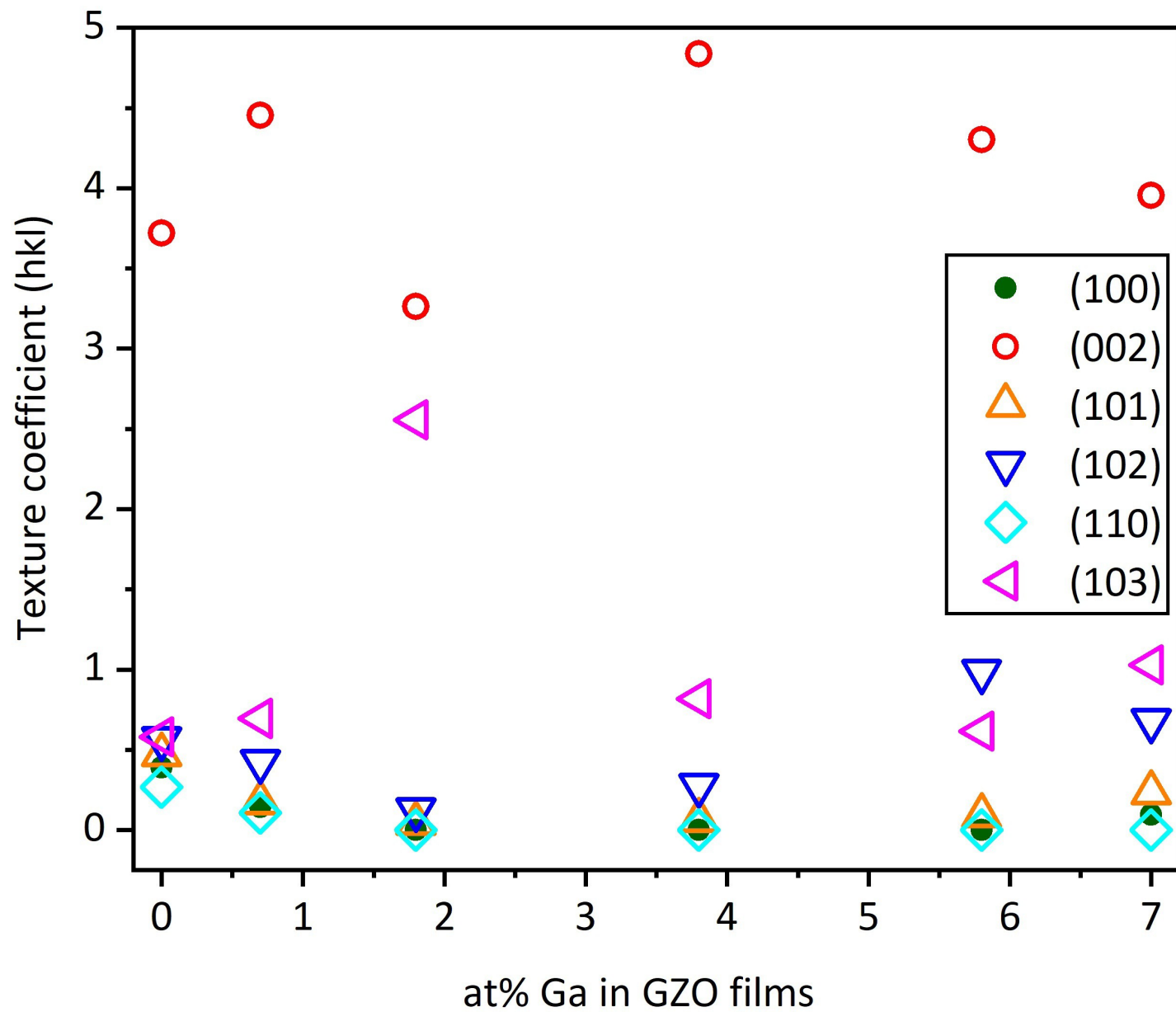

Supplement: SC-011-D0SC00502A-s014 [file SC-011-D0SC00502A-s014.pdf]
