# Supplementary material for: Aerosol-assisted route to low-E transparent conductive gallium-doped zinc oxide coatings from pre-organized and halogen-free precursor
Source: Chem Sci. 2020 Apr 27;11(19):4980–90. doi: 10.1039/d0sc00502a (PMC8159247; doi:10.1039/d0sc00502a)

Electronic Supplementary Material (ESI) for Chemical Science.
This journal is © The Royal Society of Chemistry 2020

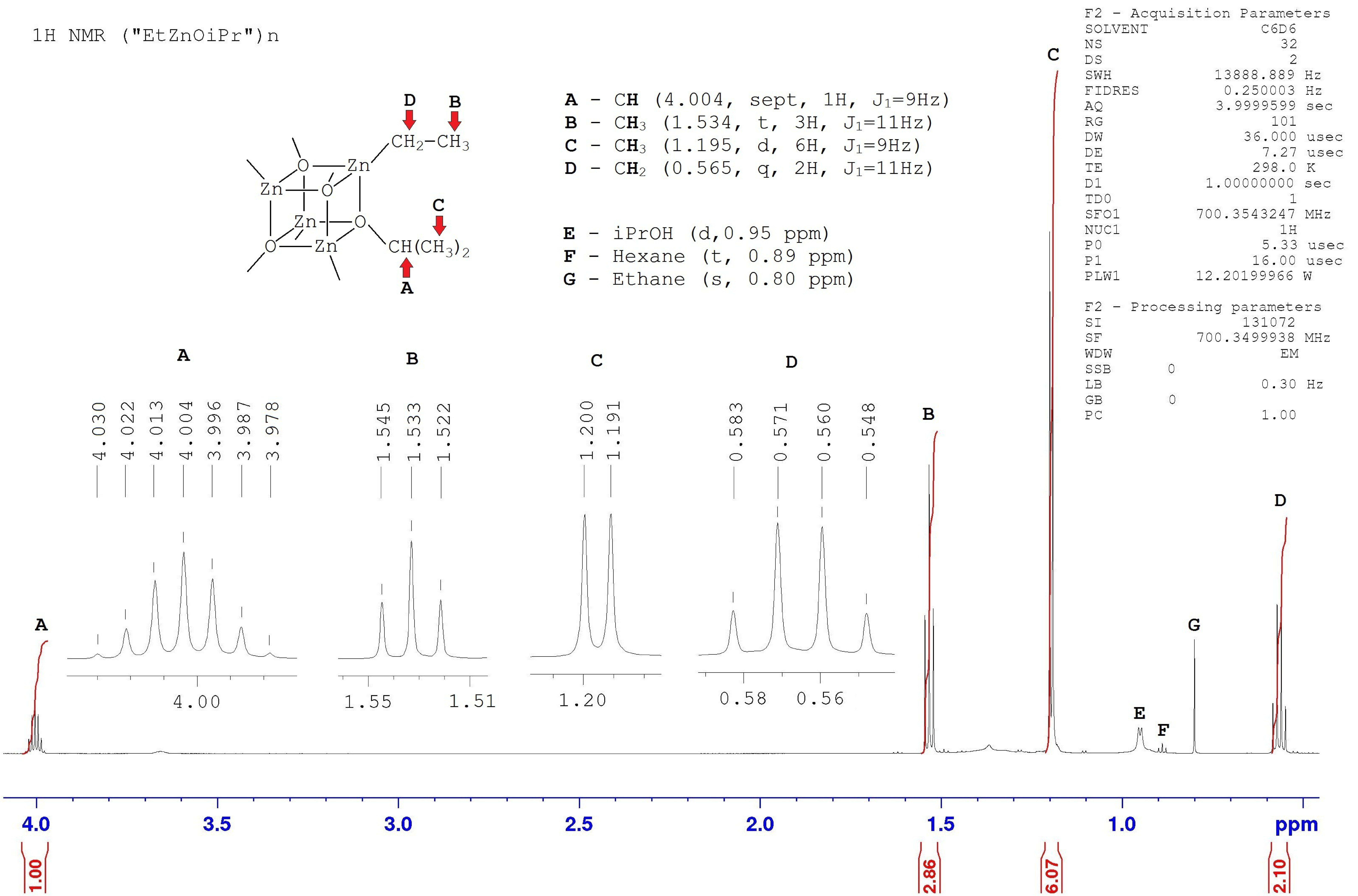

Supplement: SC-011-D0SC00502A-s006 [file SC-011-D0SC00502A-s006.pdf]
