# Supplementary material for: Aerosol-assisted route to low-E transparent conductive gallium-doped zinc oxide coatings from pre-organized and halogen-free precursor
Source: Chem Sci. 2020 Apr 27;11(19):4980–90. doi: 10.1039/d0sc00502a (PMC8159247; doi:10.1039/d0sc00502a)
Supplement: SC-011-D0SC00502A-s007 [file SC-011-D0SC00502A-s007.pdf]

Electronic Supplementary Material (ESI) for Chemical Science.
This journal is © The Royal Society of Chemistry 2020

# Aerosol-assisted route to Low-E transparent conductive gallium-doped zinc oxide coatings from pre-organized and halogen-free precursor.

Clara Sanchez-Perez, Sebastian C. Dixon, Jawwad A. Darr, Ivan P. Parkin, Claire J. Carmalt*

ELECTRONIC SUPPORTING INFORMATION

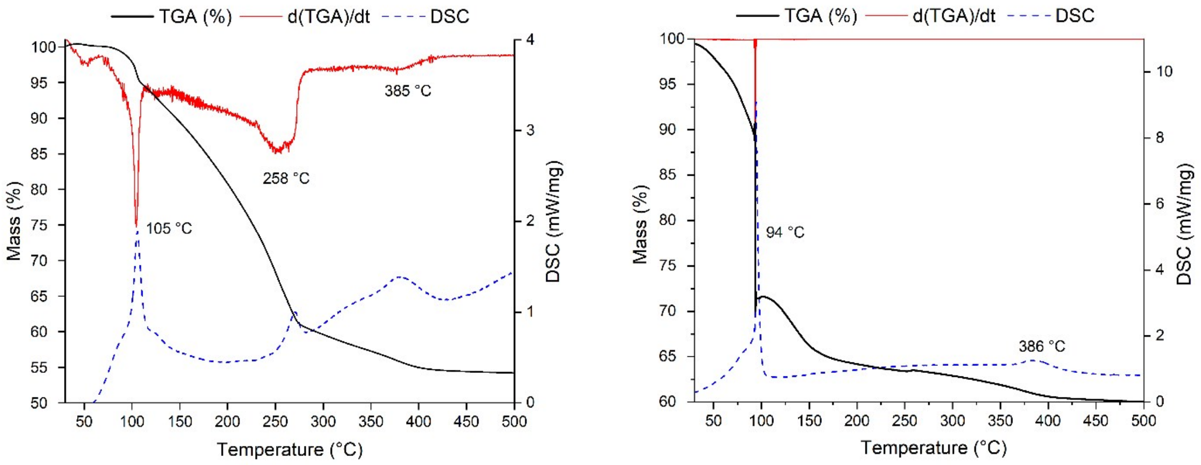


**Figure S1.** TGA(green)/DSC(blue) of precursor $[EtZnO^{i}Pr]_4$ under helium (left) and air (right). The red line represents the $1^{st}$ derivative of the mass with time.

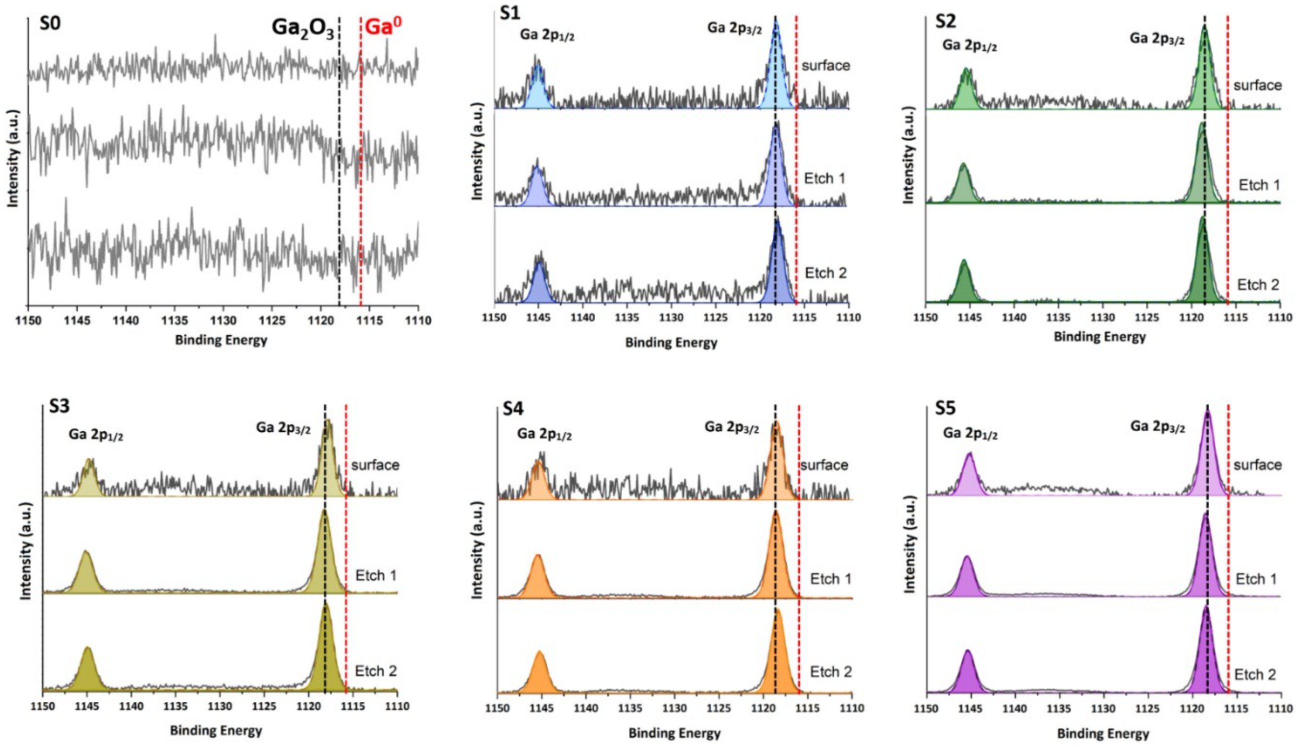


**Figure S2.** XPS scans of Ga 2p peaks of ZnO (S0) and GZO (S1-S5) thin films with increasing amounts of at% Ga. Vertical black lines indicate the literature value of Ga $2p_{3/2}$ for $Ga^{3+}$ in $Ga_2O_3$ environment and vertical red lines indicate the literature value of Ga $2p_{3/2}$ for $Ga^0$ (metal).[1]

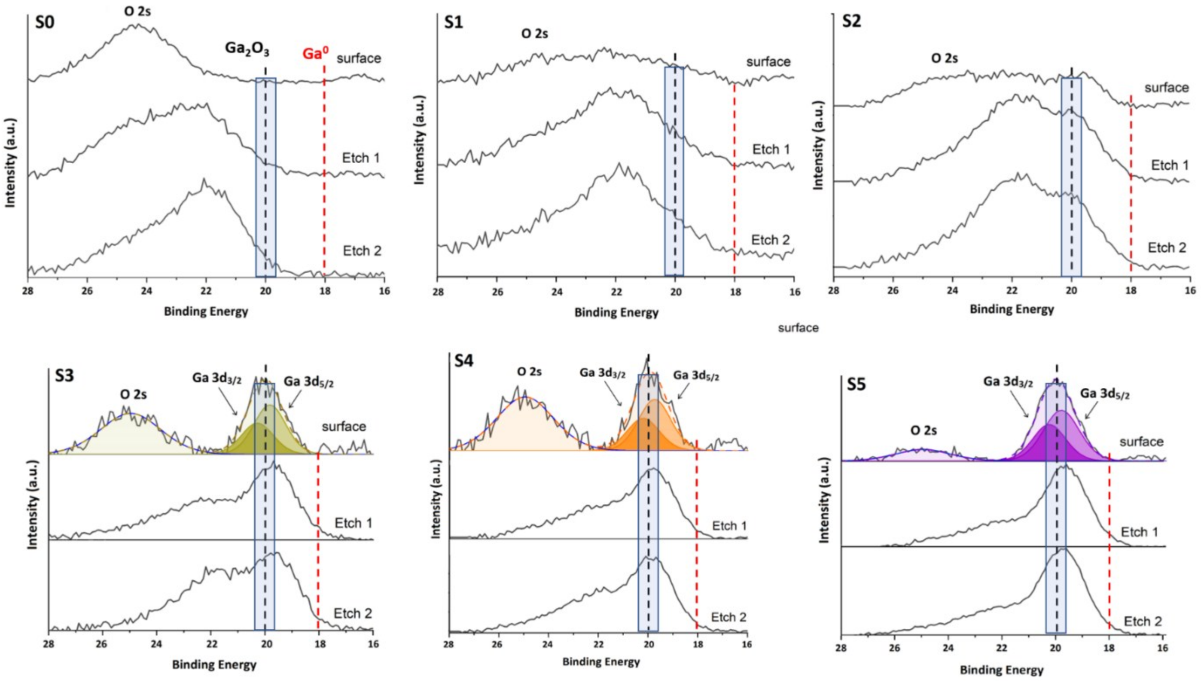


**Figure S3.** XPS scans of Ga 3d peaks of ZnO (S0) and GZO (S1-S5) thin films with increasing amounts of at% Ga. Vertical black lines indicate the literature value of Ga $3d_{5/2}$ for $Ga^{3+}$ in $Ga_2O_3$ environment[2] (blue rectangle shows error of 0.2 eV) and vertical red lines indicate the literature value of Ga $3d_{3/2}$ for $Ga^0$ (metal).[1]

**Equation S1.** Equation used to calculate the texture coefficient for each plane

$$TC(hkl) = [I(hkl)/I_0(hkl)] \,/\, [(1/N)*\sum\{I(hkl)/I_0(hkl)\}]$$

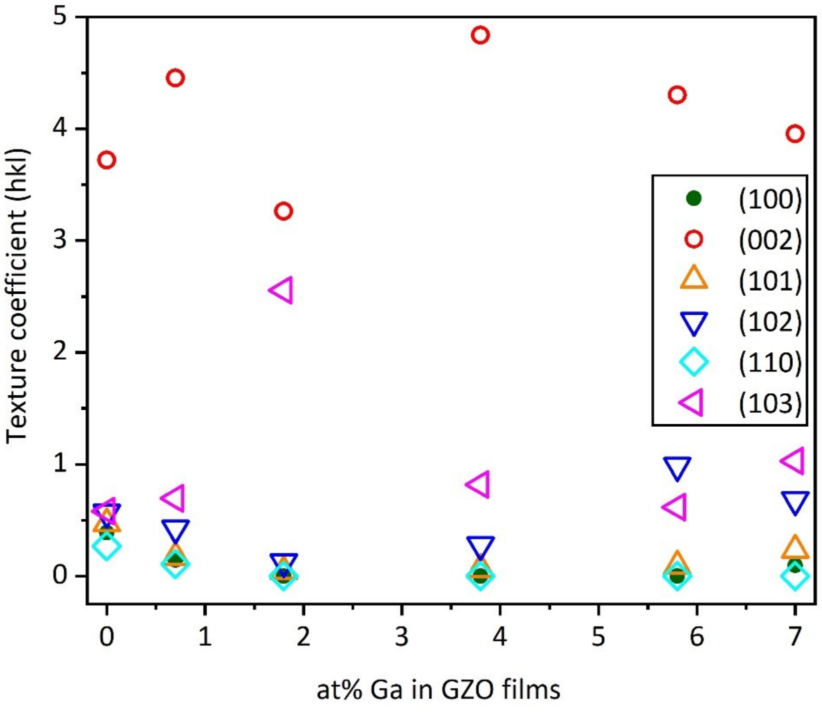


**Figure S4.** Texture coefficients for ZnO and GZO films with 0.7 – 7.0 at% Ga.

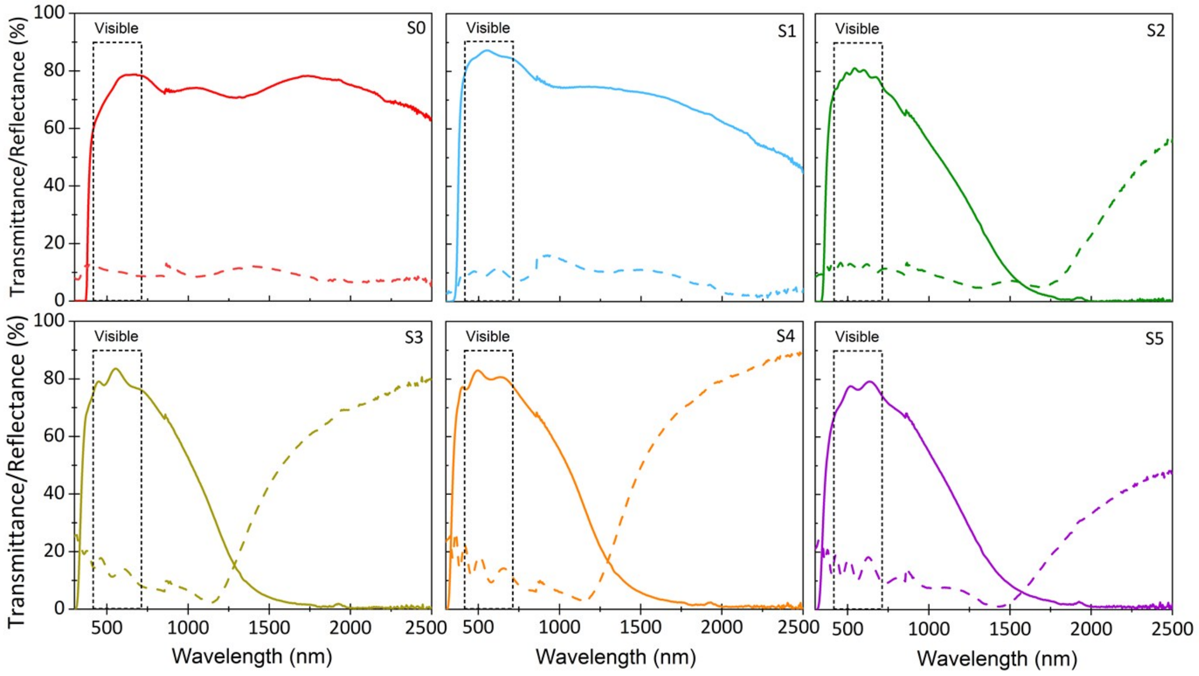


**Figure S5.** Optical transmission and reflection spectra of undoped ZnO (S0) and gallium-doped ZnO thin films with increasing at% of Ga (S1 – S5).

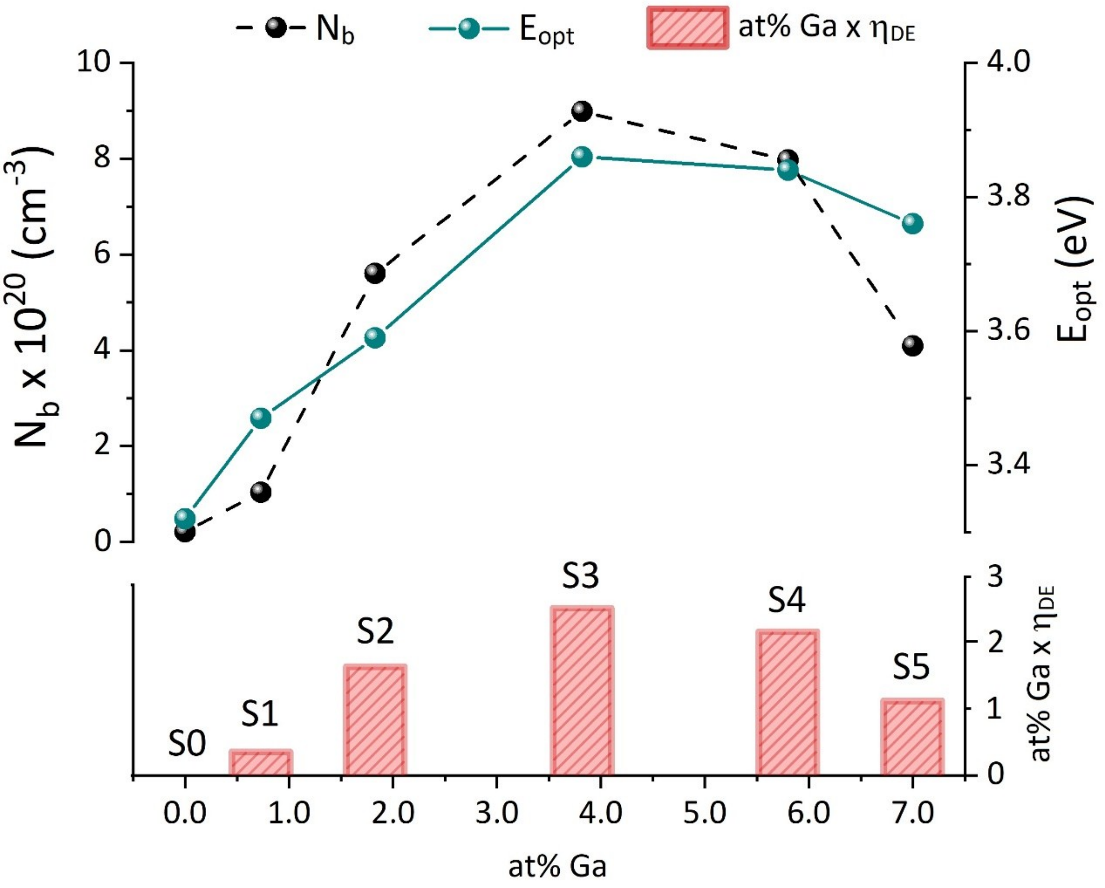


**Figure S6.** Relationship between carrier concentration ($N_b$) and band gap enhancement of GZO, in comparison with total amounts of gallium incorporated as dopant (at% Ga x $\eta_{DE}$) for each level of at% Ga.

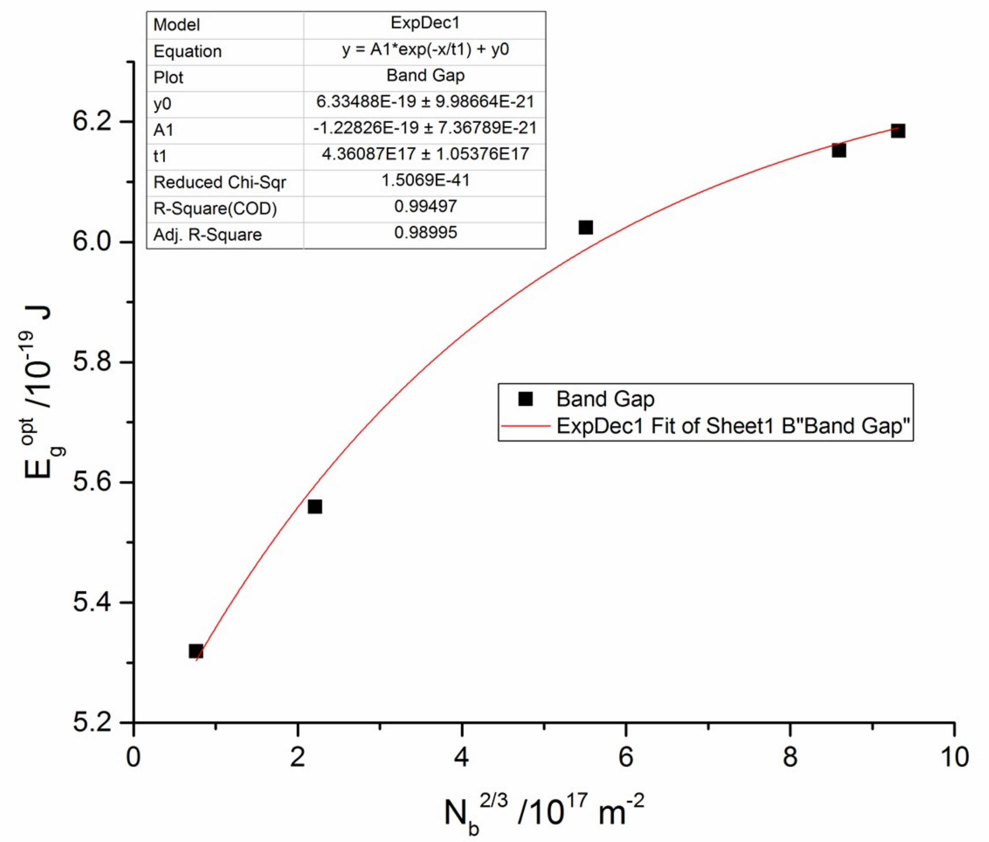


**Figure S7.** Representation of $E_g$ vs $N_b^{2/3}$ for thin films S0 – S5.

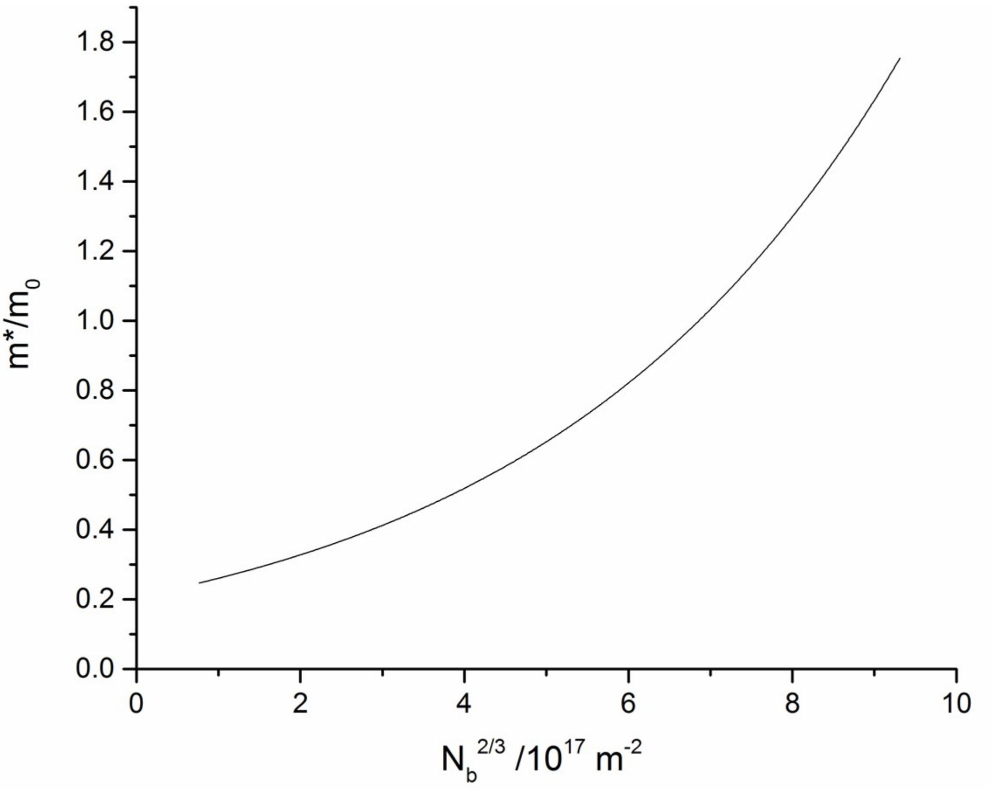


**Figure S8.** Representation of effective mass $m^*/m_0$ vs. $N_b^{2/3}$ for thin films S0 – S5.

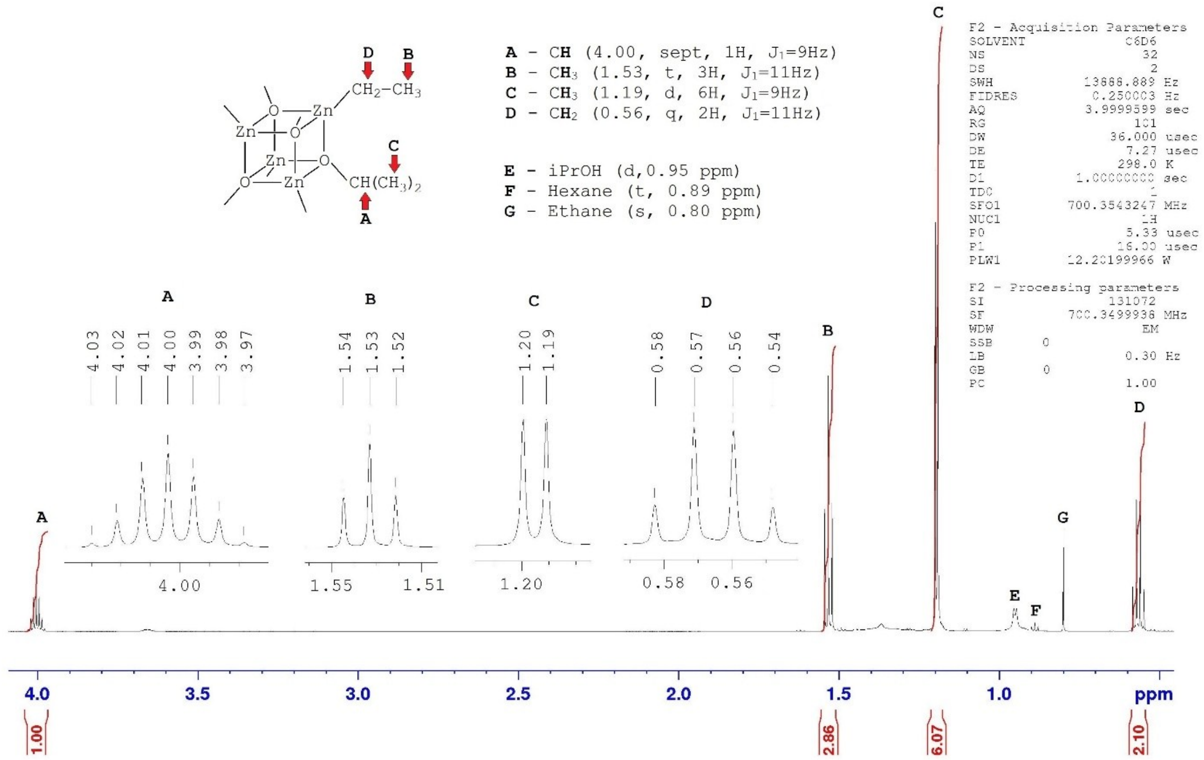


**Figure S9.** $^{1}H$ NMR of precursor $[EtZnO^{i}Pr]_4$ in $C_6D_6$.

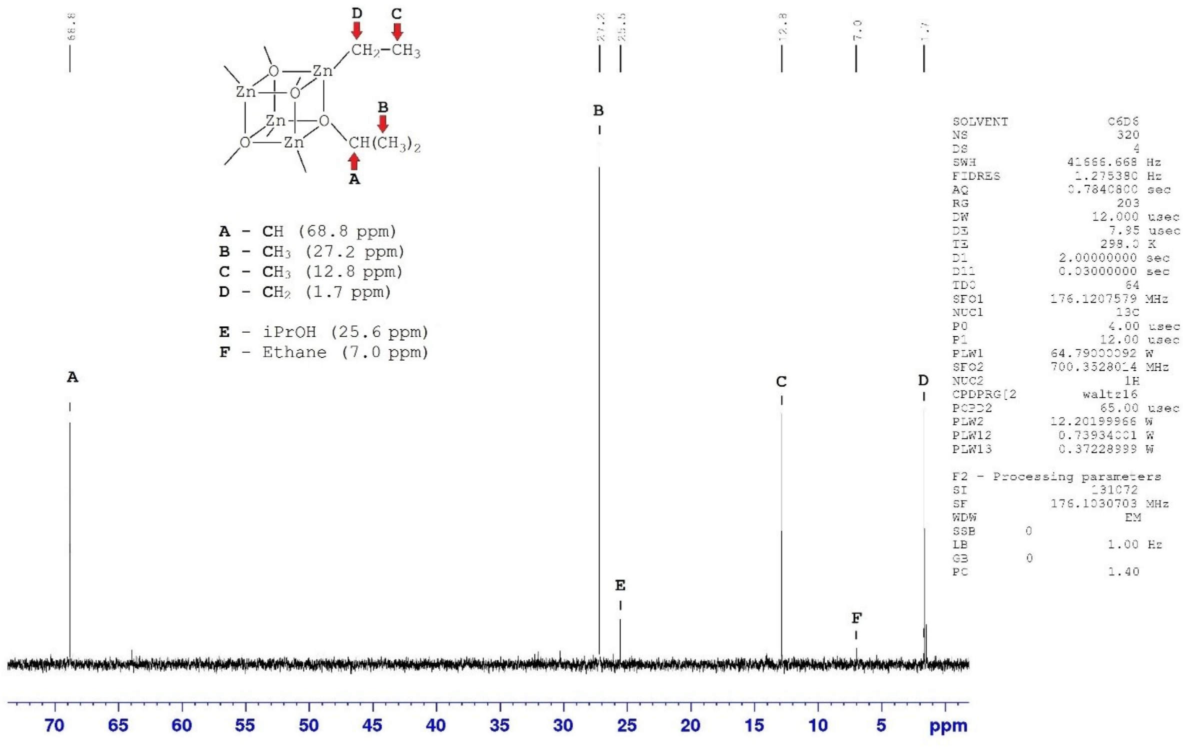


**Figure S10.** $^{13}C\{^{1}H\}$ NMR of precursor $[EtZnO^{i}Pr]_4$ in $C_6D_6$.

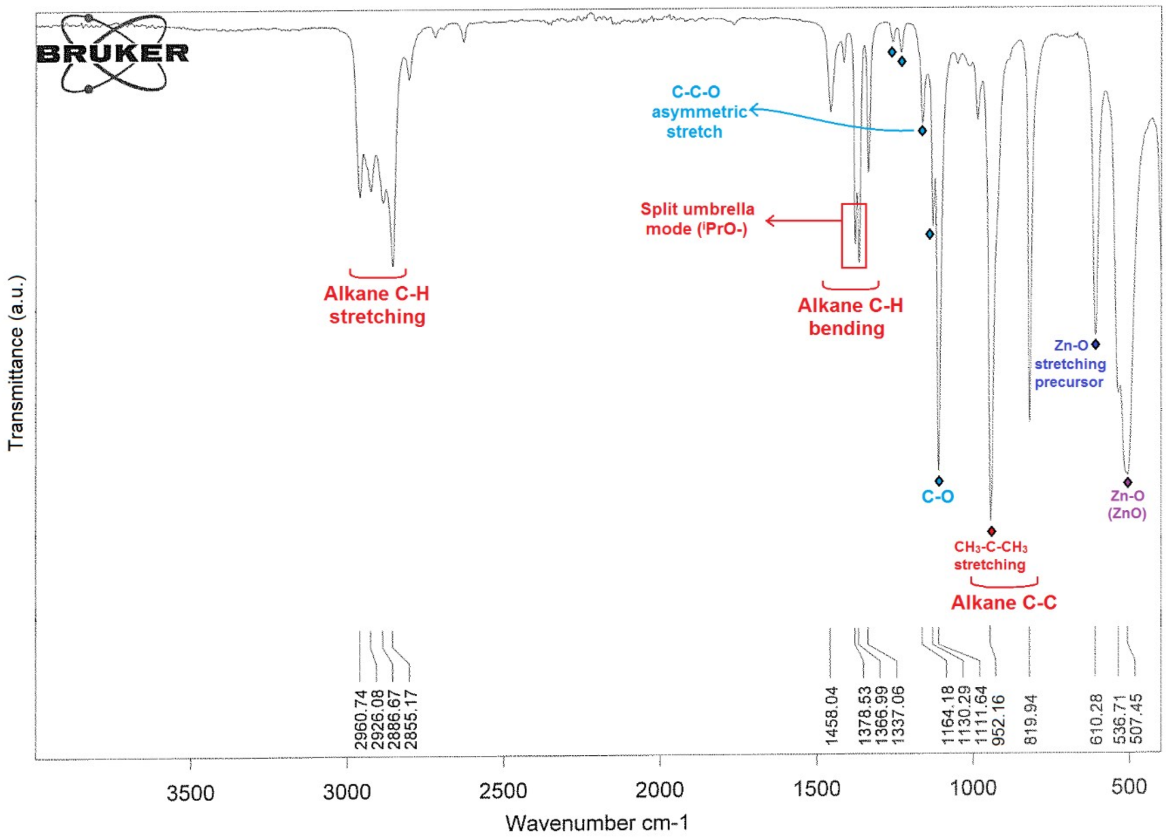


**Figure S11.** FTIR of precursor $[EtZnO^{i}Pr]_4$.

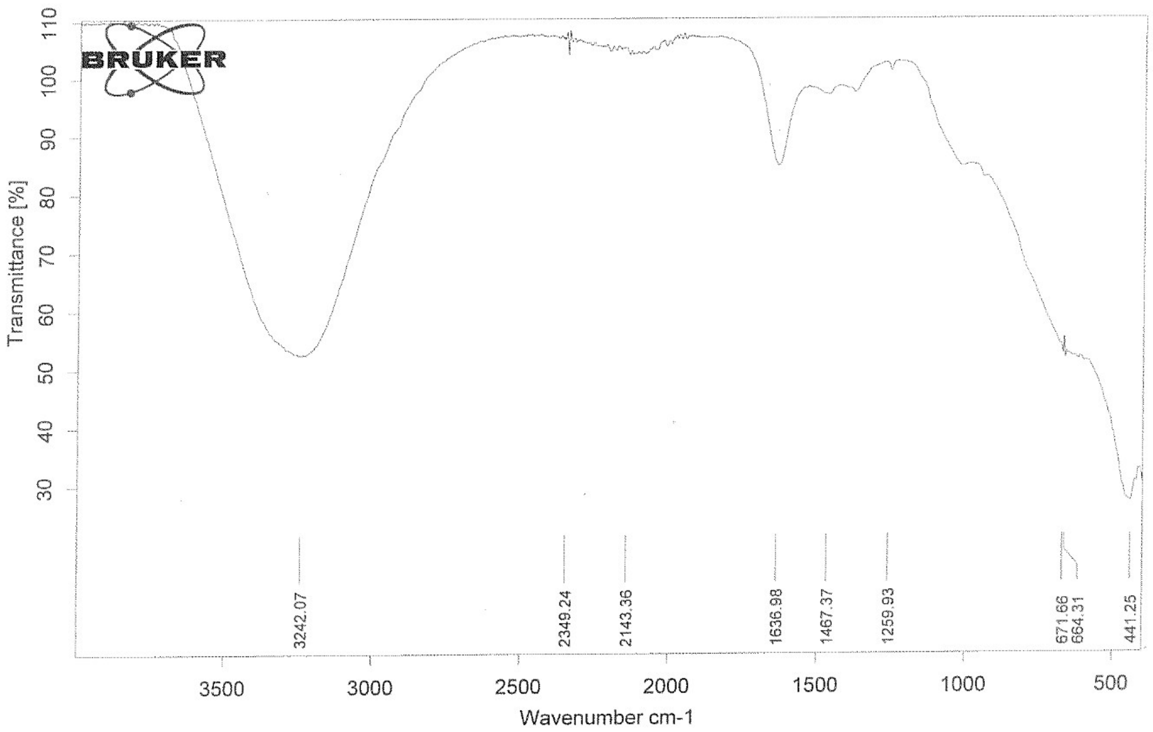


**Figure S12.** FTIR of hydrolysed precursor $[EtZnO^{i}Pr]_4$.
